# Rapid-format recombinant antibody-based methods for the diagnosis of *Clostridioides difficile* infection: Recent advances and perspectives

**DOI:** 10.3389/fmicb.2022.1043214

**Published:** 2022-11-29

**Authors:** Hamideh Raeisi, Masoumeh Azimirad, Hamid Asadzadeh Aghdaei, Abbas Yadegar, Mohammad Reza Zali

**Affiliations:** ^1^Foodborne and Waterborne Diseases Research Center, Research Institute for Gastroenterology and Liver Diseases, Shahid Beheshti University of Medical Sciences, Tehran, Iran; ^2^Basic and Molecular Epidemiology of Gastrointestinal Disorders Research Center, Research Institute for Gastroenterology and Liver Diseases, Shahid Beheshti University of Medical Sciences, Tehran, Iran; ^3^Gastroenterology and Liver Diseases Research Center, Research Institute for Gastroenterology and Liver Diseases, Shahid Beheshti University of Medical Sciences, Tehran, Iran

**Keywords:** *Clostridioides difficile*, recombinant antibody, phage display, immunoassays, immunosensors

## Abstract

*Clostridioides difficile*, the most common cause of nosocomial diarrhea, has been continuously reported as a worldwide problem in healthcare settings. Additionally, the emergence of hypervirulent strains of *C. difficile* has always been a critical concern and led to continuous efforts to develop more accurate diagnostic methods for detection of this recalcitrant pathogen. Currently, the diagnosis of *C. difficile* infection (CDI) is based on clinical manifestations and laboratory tests for detecting the bacterium and/or its toxins, which exhibit varied sensitivity and specificity. In this regard, development of rapid diagnostic techniques based on antibodies has demonstrated promising results in both research and clinical environments. Recently, application of recombinant antibody (rAb) technologies like phage display has provided a faster and more cost-effective approach for antibody production. The application of rAbs for developing ultrasensitive diagnostic tools ranging from immunoassays to immunosensors, has allowed the researchers to introduce new platforms with high sensitivity and specificity. Additionally, DNA encoding antibodies are directly accessible in these approaches, which enables the application of antibody engineering to increase their sensitivity and specificity. Here, we review the latest studies about the antibody-based ultrasensitive diagnostic platforms for detection of *C. difficile* bacteria, with an emphasis on rAb technologies.

## Introduction

*Clostridioides difficile*, an anaerobic Gram-positive spore-forming bacillus, is a medically important pathogen and known as the main cause of diarrhea in humans globally (Burke and Lamont, 2014). *C. difficile* can asymptomatically be present in the gut of healthy individuals (Furuya-Kanamori et al., 2015) or lead to infections with a wide spectrum of clinical disorders, including abdominal pain, diarrhea, pseudomembrane colitis (PMC), or even in some cases death (Burke and Lamont, 2014; Orrell and Melnyk, 2021). Currently, *C. difficile* infection (CDI) is identified as the major cause of nosocomial diseases associated with antibiotic therapy (in particular cephalosporins, clindamycin, metronidazole, and vancomycin) and healthcare-associated diarrhea in adults (Viswanathan et al., 2010; Cornely et al., 2012; Azimirad et al., 2020a, 2022). Additionally, other risk factors are involved in CDI incidence, including immunosuppression, previous hospitalization, age above 65 years, and the use of proton pump inhibitors (Surawicz et al., 2013; Eze et al., 2017; Azimirad et al., 2021). Notably, the spore-forming nature of *C. difficile* can be paired with its ability to rapidly colonize the intestine of patients and arises a critical challenge in infection control and treatment in both the community and healthcare settings (Paredes-Sabja et al., 2014; Castro-Córdova et al., 2021). In the last two decades, the number of CDI patients has been increasing (Depestel and Aronoff, 2013; Azimirad et al., 2020b; Baghani et al., 2020), so that in the USA, *C. difficile* imposes more than 453,000 illnesses per year, leading to 29,600 deaths. These estimates confirm that *C. difficile* is a major continuous burden to public health (Lessa et al., 2015; Kelly et al., 2021). On the other hand, CDI treatment contributed to substantial healthcare cost, and in the USA alone the cost associated with CDI management exceed $4.8 billion annually (Dubberke and Olsen, 2012; Balsells et al., 2016). Additionally, antibiotic therapy for CDI can result in further disruption of the normal gut microbiome and the development of hypervirulent strains of *C. difficile*. Alternatively, 20–30% of the patients with primary infection symptoms experience recurrent disease within 2–6 weeks after the completion of antibiotic treatment (Cornely et al., 2012; Raeisi et al., 2022a), which would be higher after secondary and tertiary CDI and more refractory to treatment regimens (Cornely et al., 2012; Eze et al., 2017). Therefore, rapid and accurate diagnosis of CDI has been a troublesome issue for disease management, implementation of infection control measures, and epidemiological monitoring (Burnham and Carroll, 2013; Peng et al., 2018). Additionally, an early and efficient diagnosis can positively impact the clinical outcome of CDI patients with primary infection and reduce the rate of infection recurrence (Barbut et al., 2014).

In recent years, antibodies, i.e., polyclonal (pAb) and monoclonal antibodies (mAbs), have been extensively applied for diagnostic and therapeutic purposes of various human diseases and cancers (Roovers et al., 2011; Ascoli and Aggeler, 2018; Haji-Hashemi et al., 2018; Raeisi, 2018; Raeisi et al., 2020; Zahavi and Weiner, 2020; Chiari et al., 2021; Hwang et al., 2022). In this regard, antibodies are exploited due to their high affinity and specific binding activity toward target molecules, which highlights their effectiveness as theranostics tools. In recent decades, recombinant antibody (rAb) technologies have widely received much attention as diagnostic tools (Xu et al., 2017; Shali et al., 2018; Fouladi et al., 2019; Raeisi et al., 2019; Alibeiki et al., 2020; Raeisi et al., 2020, 2022b; Berry et al., 2022). The rAb technologies that involve the construction of large libraries of different antibody fragments, such as fragment antigen-binding (Fab), single-chain fragment variable (scFv), minibodies, and nanobodies, can screen antibodies *in vitro* based on their binding properties, thus help develop more cost-effective antibodies with high sensitivity and specificity (Bockstaele et al., 2000; Angela Chiew Wen et al., 2016; Raeisi et al., 2022). This review will focus on currently available procedures for CDI diagnosis and different antibody-based rapid detection methods with an emphasis on rAbs. Additionally, analytical aspects of rAbs as recognition elements to develop ultrasensitive methods will be discussed.

## Conventional techniques for *Clostridioides difficile* detection

Some of the major and common *C. difficile* antigens are its surface proteins, including surface-layer proteins (SLPs), cell wall protein 66 (Cwp66) and 84 (CWP84), flagellin FliC, flagellar cap protein FliD, which play key role in attachment to intestinal mucus and have been used as target biomolecules in previous studies (Merrigan et al., 2013; Kandalaft et al., 2015; Shirvan and Aitken, 2016). Additionally, glutamate dehydrogenase (GDH) is a constitutive enzyme produced in large amounts by all toxigenic and non-toxigenic strains of *C. difficile* and can be easily detected in stool samples (Arimoto et al., 2016; Kelly et al., 2021). However, the introduction of toxin-producing strains of *C. difficile*, as the main cause of antibiotic-associated diarrhea (AAD) in the 1970s, led to the application of several diagnostic methods to detect CDI, most of which relying on the detection of toxin A (TcdA) or B (TcdB) (Kelly et al., 2021). In fact, the main symptoms of CDI are contributed to the secretion of bacterial toxins (Di Bella et al., 2016) that affect the epithelial cells of the gastrointestinal (GI) tract by inactivating Rho/Ras proteins and subsequently lead to loss of epithelial barrier function, cytoskeleton disintegration, condensation of actin, severe inflammation, and eventually cell death (Chen et al., 2015a; Di Bella et al., 2016). These outcomes damage the patient’s colonic mucosa and cause severe diarrhea and PMC. Some strains also express an additional toxin, named binary toxin CDT, but its role in the CDI pathogenesis has not been fully understood yet (Chandrasekaran and Lacy, 2017). Notably, the majority of the studies have demonstrated that only toxigenic strains, producing TcdA and/or TcdB, are pathogenic and can cause CDI (Di Bella et al., 2016; Chandrasekaran and Lacy, 2017). Moreover, based on the criteria declared by the European Society of Clinical Microbiology and Infectious Diseases (ESCMID), a CDI case is diagnosed when there are clinical symptoms compatible with CDI (usually diarrhea), stool test positive for TcdA and/or TcdB of *C. difficile* without evidence of another cause of diarrhea, and colonoscopy or histopathological data revealing PMC (van Prehn et al., 2021).

Currently, various methods have been introduced for detecting *C. difficile*, the most important of which being toxigenic culture (TC), cell-culture cytotoxicity neutralization assay (CCNA), enzyme immunoassay (EIA) for TcdA and/or TcdB, GDH detection, polymerase chain reaction (PCR), and stool culturing (Kelly et al., 2021). However, these diagnostic methods have several limitations in terms of speed, sensitivity, specificity, cost and ease-of-use (Gite et al., 2018). Generally, TC and CCNA are considered as reference methods for *C. difficile* identification by detecting toxin A and/or B (Planche and Wilcox, 2011). The application of the TC assay to stool samples leads to the isolation of *C. difficile* strains and determination of their ability to produce toxins. Although this test has shown high sensitivity (95%), it is very cumbersome, expensive, and time-consuming as it takes 3–5 days to be completed (Yücesoy et al., 2002). In addition, TC assays alone may show false-positive results due to the presence of non-toxigenic strains (Alcalá et al., 2008). On other hand, CCNAs have also shown high sensitivity and specificity (90–95%) for detecting toxigenic *C. difficile* strains, but these tests examine the production of toxins *in vitro*, which may not reveal correlations between fecal toxin levels and disease severity. In fact, *in vitro* examination may not reflect the actual *in vivo* toxin levels (Pollock, 2015).

The limitations of using cytotoxicity assays have led to the introduction of alternative techniques, including the application of numerous commercial qualitative EIA tests for detecting toxins A or/and B. These techniques have high specificity (96–98%) compared to toxigenic culture, but low sensitivity (52–75%) (Eastwood et al., 2009; Moon et al., 2016). Therefore, nucleic acid detection techniques that are highly sensitive, should be used as a complementary method (Burnham and Carroll, 2013). However, nucleic acid amplification tests (NAATs) alone are not recommended because these tests can lead to false-positive results as they detect toxin-encoding genes of *C. difficile* regardless of toxin production (Gateau et al., 2018). Recently, detection of pathogen-specific proteins like GDH by EIA or latex particles has been proposed in some studies (Davies et al., 2015; Yoldas et al., 2016). GDH is easily detectable in stool samples and also known as a good screening marker for *C*. *difficile*. GDH detection methods are of high sensitivity but low specificity as GDH is highly abundant in both toxigenic and non-toxigenic strains, as a result, these methods are incapable to differentiate CDI from asymptomatic colonization or the presence of non-toxigenic strains (Surawicz et al., 2013), thus, additional EIA for detection of toxins A, B, or both is required (Badger et al., 2012; Kelly et al., 2021). A summary of the specificity and sensitivity of various diagnostic tests for CDI compared to toxigenic culture are presented in Table 1.

**Table 1:** Summary of diagnostic tests for *Clostridioides defficile* infection.

**Test**	**Substance detected**	**Sensitivity %**	**Specificity %**	**Time required**	**Limitations**	**Reference**
TC	*C. difficile*	95	80–90	3–5 days	Long turnaround time, trained personnel	Davies et al. (2009), Moon et al. (2016)
CCNA	Toxin	95	90–95	1–3 days	Long turnaround time, trained personnel, technical demands	Huang et al. (2009), Reller et al. (2010)
GDH	*C. difficile*	95–100	88–92	Hours	Low specificity, false-negative results	Eastwood et al. (2009), Cheng et al. (2015), Ramos et al. (2020)
EIA toxin	Toxin	51–80	98–99	Hours	Low sensitivity	Davies et al. (2009), Eastwood et al. (2009)
NAATs	*C. difficile*	92–97	83–100	Hours	Low specificity, costly and technical demands	Davies et al. (2009), Eastwood et al. (2009), Paitan et al. (2017)
GDH and EIA toxin	Toxigenic *C. difficile*	83–100	97–100	Hours	Dependent on toxin results, some variation in reported sensitivity	Kim et al. (2014), Cheng et al. (2015)
NAATs and EIA toxin	Toxigenic *C. difficile*	77–100	91–100	Hours	Some variation in reported sensitivity, technical demands	Huang et al. (2009), Humphries et al. (2013)

TC, toxigenic culture; CCNA, cell-culture cytotoxicity neutralization assay; EIA, enzyme immunoassay; GDH, glutamate dehydrogenase; NAATs, nucleic acid amplification tests.

**Figure 1:**
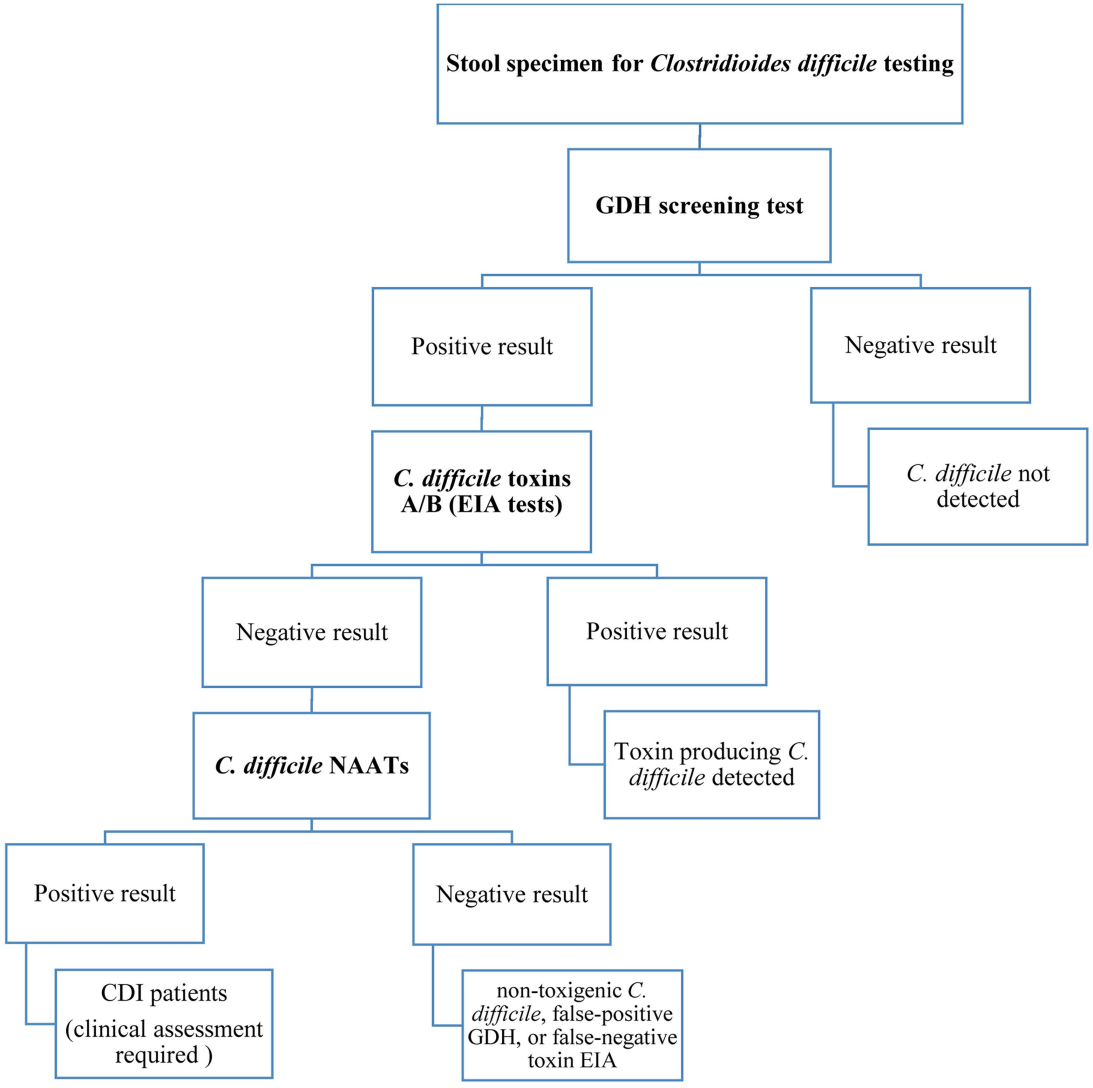
Three-step algorithm for diagnosis of *Clostridioides difficile* infection.

Presently, there is no agreement on the most appropriate laboratory tests used for CDI diagnosis but much clinical reliance is still on the detection of TcdA and/or TcdB by enzyme-linked immunosorbent assay (ELISA) platforms (Moon et al., 2016; Yoldas et al., 2016; Paitan et al., 2017). However, it is still controversial whether to give priority to detecting bacterial toxins or bacterial infection. Several studies have shown a significant correlation between the level of toxins in the stool and disease severity, as a result, determining the toxin concentrations in the stool can be helpful in disease management and predicting treatment outcomes (Planche et al., 2013; Di Bella et al., 2016; Pollock et al., 2019). Importantly, when the prevalence of *C. difficile* infection is low, no diagnostic test should be used alone because of low positive predictive values. Besides, dissimilarity of the results obtained from different diagnostic tests has made it difficult to consent to a single and reliable standard method, thus, the application of a simple and rapid technique with high sensitivity and specificity for *C. difficile* detection is challenging yet (Burnham et al., 2015; Song et al., 2015). Altogether, the diagnosis of CDI is often confirmed based on the positive results of two or three detection tests (Oldfield et al., 2014), and it is generally recommended to use the three-step algorithm as shown in Figure 1 for accurate CDI diagnosis.

In the past two decades, more rapid methods for detection of toxins of *C. difficile* have been developed and some of them are highly rapid and sensitive but are not yet commercially available (Jarrige et al., 2011; Pollock, 2015; Wilson et al., 2015; Gite et al., 2018). However, providing desirable performance characteristics such as being rapid, easy-to-use, and cost-effective can lead to clinical application of these emerging methods. So far, two commercial clinical biosensor kits have been introduced for CDI diagnosis that can detect the infection in a very short time (Table 2). Additionally, a wide variety of antibody-based diagnostic methods are available for detecting infections of the GI tract. Some of these methods, such as immunofluorescence or ELISA, are laboratory techniques and need trained personnel, while others, such as agglutination techniques, membrane EIAs, and lateral flow immunoassays (LFIAs), are very simple and fast (Balsalobre-Arenas and Alarcón-Cavero, 2017). Different formats of antibodies applied in diagnostic tools are discussed below.

**Table 2:** Examples of commercially available clinical biosensors kits for *Clostridioides difficile* infection.

**Platform**	**Target (analyte)**	**Transducer Platform Biosensor**	**Detection format**	**Applicability**	**LOD**	**Response time**	**Reference**
Commercially available clinical biosensors	GDH (Clostridioides *K*-SeT)	Membrane-based lateral flow assay	Optical	Stool	0.5 ng/mL	15 min	https://www.corisbio.com/products/Clostridioides-k-set
	Whole cells (DR1107A, Oxoid, Hampshire, UK).	Rapid latex agglutination	Colorimetric latex agglutination	Selective media	N/A	2 min	https://www.thermofisher.com/order/catalog/product/DR1107A#/DR1107A

GDH, glutamate dehydrogenase; N/A, not available.

## Applicable formats of antibody for detection purposes

**Figure 2:**
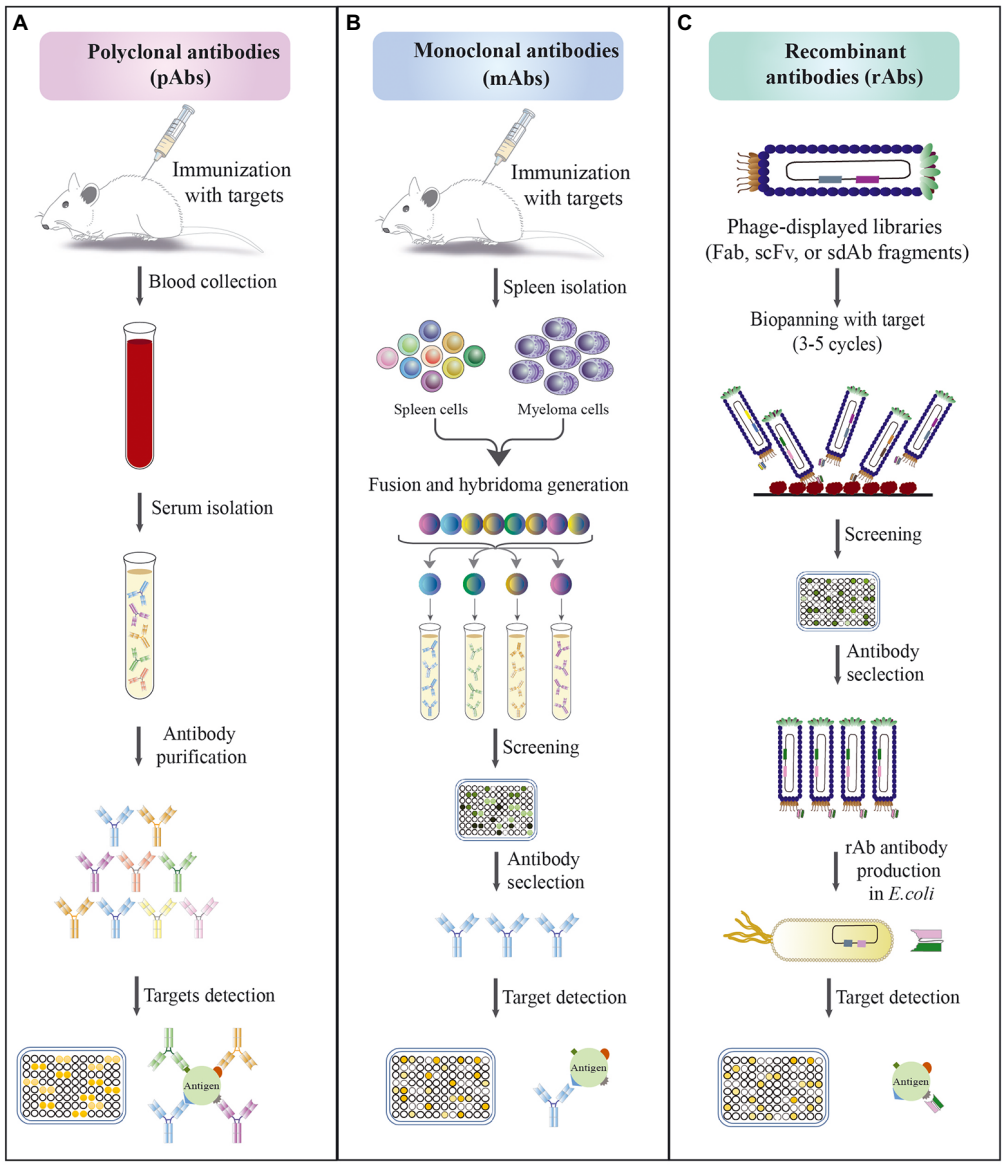
Schematic approaches for the development of different types of antibodies. **(A)** Polyclonal antibodies production; the methods’ steps include: (1) immunization of animals with an antigen to trigger immune response; (2) blood collection from immunized animals; (3) serum isolation; (4) antibody purification; (5) evaluation of the affinity of antibodies against antigen by immunoassay. **(B)** Monoclonal antibody production by hybridoma technique; the methods’ steps include: (1) immunization of animals with an antigen to trigger immune response; (2) isolation of antibody-producing cells from the mouse spleen; (3) fusion of B cells with myeloma cells to produce hybridoma cells; (4) screening and selection of high affinity antibody produced by hybridoma cells; (5) evaluation of the specificity of selected antibodies against antigen by immunoassay. **(C)** Recombinant antibody production by phage display technology; the methods’ steps include: (1) amplification of library; (2) exposure of the library to a surface coated with specific antigen; (3) elution of bound phages and their application for a new selection round; (4) screening of positive phage clones by ELISA after 3–5 rounds of biopanning; (5) selection of specific phages and their expression; (6) evaluation of the specificity of selected antibodies against antigen by immunoassay.

Antibody-based detection methods, like EIA, ELISA, and LFIAs or immune-chromatographic tests (LFIA/ICT), are mostly designed based on the use of mAbs and pAbs in their platforms. The antibodies were first produced as pAbs and their production is still ongoing for various purposes. Normally, pAbs are made through the vaccination of animals such as rabbits, goats, and sheep, and can be rapidly generated at less expense and not requiring complicated technical skills (Stills, 2012). These molecules are produced by different B cell clones in the body, thus are usually a heterogeneous mixture, which can recognize multiple epitopes of antigen (Figure 2A). This property of pAbs can lead to the risk of low specificity and cross-reactivity with different targets (Ascoli and Aggeler, 2018).

The technique of mAb production, known as hybridoma technology, was invented by Kohler and Milstein in 1975 (Köhler and Milstein, 1975; Figure 2B). Although the hybridoma technology has been successful, it has many shortcomings, e.g., limited number of candidates, time-consuming, inefficiency to generate antibodies toward highly conserved antigens, self-antigens, sensitive antigens, and toxic antigens. A strategy that overcome the disadvantages of hybridoma is techniques based on *in vitro* antibody production, e.g., yeast display, ribosome display, phage display, and mammalian cell display methods (Raeisi et al., 2022a; Valldorf et al., 2022), which among them, phage display and ribosome display are preferred in most laboratories due to their large diversity in the range of 10^12^–10^15^ variants with high transformation efficiency (Kunamneni et al., 2020; Raeisi et al., 2022a). Here, we discuss the rAb techniques that are currently being studied for diagnosis proposes.

### *In vitro* recombinant antibody technologies

*In vitro* antibody display technologies, also called recombinant antibody technology, are prepared based on the diverse antibody genes contained in a specified library. The first display technology introduced was phage display, which was presented by Georg P. Smith in 1985 (Willats, 2003). Since these technologies depend on *in vitro* screening rounds, the production of antibodies could be much easier and more cost-effective than *in vivo* conditions (Alfaleh et al., 2020). A unique property of rAbs is the high affinity and specificity of them for detecting target molecules. Alternatively, the main concern in the development of immunoassays has always been the increase in the sensitivity and affinity of antibodies, which can be achieved by the possibility of integration of *in vitro* antibody display technologies with genetic engineering (83). In addition, using *in vitro* expression systems for the production of rAb fragments can finally lead to achieving sufficient amounts of antibodies for diagnostic purposes (Wang et al., 2013; Jensen et al., 2018) and allowing the construct of new different types of rAb fragments, including Fab, scFv, domain antibodies (dAbs), bivalent antibodies, multivalent antibodies, and bispecific antibodies (Yang et al., 2014; Fan et al., 2015; Høydahl et al., 2016). In terms of production of rAbs, ribosome display and phage display technologies have been introduced as potent *in vitro*, cell-free systems for the screening of large libraries, which can select high-affinity binders against various antigens without compromising the library (Li et al., 2019; Hammerling et al., 2020; Valldorf et al., 2022).

### Ribosome display and phage display technologies

Ribosome display technology is known as a powerful tool for the rapid isolation and direct evolution of high-affinity binders, particularly antibodies. This technology generates a stable complex containing antibody–ribosome–mRNA that can link individual antibody fragments to their respective mRNA (Kunamneni et al., 2020). Conceptually, the ribosome display technique includes several steps, including library preparation, *in vitro* transcription and translation, screening and selecting a specific mRNA, and reverse transcription-PCR (RT-PCR) for further screening rounds or analysis (Li et al., 2019). Briefly, in this method, the mRNA lacks a termination codon (nonstop mRNA) at the end of the coding sequence, which holds the translating ribosome at the end of mRNA and creates an unreleased nascent polypeptide. This process results in the formation of antibody–ribosome–mRNA complexes, allowing simultaneous isolation of antibody fragments and their corresponding mRNA through an affinity for an immobilized antigen. To isolate high-affinity protein–mRNA complexes, 3–5 rounds of panning are carried out, and selected complexes are subjected to *in situ* RT-PCR to recover the DNA encoding protein sequence (Kunamneni et al., 2020).

Within the past two decades, the production of rAbs, particularly scFvs, has been strikingly accelerated through ribosome display technology. Moreover, this technology has become a popular tool in medicine for basic research, disease diagnostics, and therapy (Park, 2020; Valldorf et al., 2022). However, one of the limitations of this method is the accessible, functional ribosome levels in the reaction for the library, which relates to the library size (Kunamneni et al., 2020). The ribosome display technology has been applied for selecting scFvs with high affinity against different targets such as tumor cells (Huang et al., 2018), Zika virus (ZIKV) (Kunamneni et al., 2018), *Mycobacterium tuberculosis* (Ahangarzadeh et al., 2017), and severe acute respiratory syndrome coronavirus 2 (SARS-CoV-2) (Chen et al., 2021). Moreover, it has been successfully used for scaffold selection, including affibodies (Lagoutte et al., 2019), however, there are no reports about antibody selection through ribosome display for CDI diagnosis or treatment. Overall, this method could be undoubtedly considered to select specific antibodies for new targets for diagnostic or therapeutic purposes in the future.

Additionally, the phage display technique is one of the most widely used technologies for the production of rAbs. In this technology, specific peptides or proteins are displayed on the surface of the filamentous phage particles through fusion between the genes encoding the antibody with the coat protein (pIII or pVIII) of the phage (Figure 2C). Therefore, a foreign phenotype is displayed on the phage surface that it’s the genotype is retrievable in the phage (Schirrmann et al., 2011). The phage display technique contains the following steps: cloning of antibody-gene library, packaging of the resulting phagemids into phage particles presenting the respective antibody fragment on its surface, bio-panning to enrich antibodies binding to target structure, amplification of binding antibody phage, screening of individual antibody clones for the desired characteristics, e.g., by ELISA, subcloning of selected antibodies, and expression in format and expression system of choice (Hammers and Stanley, 2014). The most common method of bio-panning is coating antigens on a solid surface with high protein-binding capacity, such as polystyrene tubes (Jensen et al., 2018; Lu et al., 2020). In the next step, the surface coated by antigen is exposed to phage library, then, stringent washing of the surface is performed to remove nonbinding antibody phage. Subsequently, elution of the bound antibody phage is conducted by methods like pH shift or by using trypsin, and then, reamplification is done by infection of *Escherichia coli*. Reamplified phage will be used in the next round of panning. It is expected that after each round of panning, the number of specific binding phages is increased. After 3–5 panning rounds, antigen-specific antibody phage will be checked by several methods such as ELISA, immunoblotting, or flow cytometry (Bazan et al., 2012). This approach is similarly done for the production of both therapeutic and diagnostic antibodies (Schirrmann et al., 2011; Frenzel et al., 2017).

The phage display technique has been widely used for detecting various targets, such as haptens, toxins, foreign and self-antigens (Tullila and Nevanen, 2017; Hussack et al., 2018; Raeisi et al., 2022a). For example, a specific antibody against a tumor marker expressed by breast tumor, named ErBb2 protein, has been selected through naïve antibody library that is useful in immunoassay tools (Bakir et al., 1993). Furthermore, many rAbs derived from page libraries have been examined for diagnosis or therapy of bacterial diseases; the majority of these rAbs have been developed to facilitate disease detection and estimate the presence of contamination in environmental and food samples or levels of sample contamination (Kuhn et al., 2016; Priyanka et al., 2016). These rAbs are usually designed for various purposes, including detection of whole bacterial cells or toxins, blocking receptors, and modulating the host immune system (Kuhn et al., 2016), among them, whole bacterial cells are mostly applied for diagnostics (Byrne et al., 2013). An overview of rAbs derived from phage display libraries against *C. difficile* is presented in Table 3.

**Table 3:** Recombinant antibodies derived from phage display libraries against *Clostridioides difficile*.

**Target**	**Antibody format**	**Antibody origin**	**Application**	**References**
TcdB	scFv	Human	ELISA	Deng et al. (2003)
FliC, FliD	scFv	Human	ELISA, WB, *in vitro* assay	Shirvan and Aitken (2016)
SLP	sdAb	Llama	ELISA, WB, *in vitro* assay	Kandalaft et al. (2015)
TcdA	sdAb	Llama	ELISA, WB, *in vitro* neutralization	Hussack et al. (2011)
TcdA, TcdB	sdAb, bispecific	Alpaca	ELISA, *in vitro* assay, *in vivo* protection	Yang et al. (2014)
TcdB	scFv-Fc	Human	*in vitro* neutralization	Chung et al. (2018)
Binary CDT toxin	sdAb, sdAb-Fc	Llama	ELISA, IF	Unger et al. (2015)

TcdA, toxin A; TcdB, toxin B; SLP, surface-layer protein; scFv, single-chain fragment variable; sdAb, single-domain antibodies; Fc, fragment crystallizable region; ELISA, enzyme linked immunosorbent assays; WB, western blot; IF, immuno fluorescence microscopy.

## Development of rapid detection methods based on recombinant antibodies against *Clostridioides difficile*

In recent decades, the design of rapid diagnostic methods based on pAb or mAb antibodies have been attracted much attention. Accordingly, a wide variety of antibody-based diagnostic methods are available for the diagnosis of GI tract infections, such as immunofluorescence, ELISA, and various immunosensors. Additionally, the rAb technologies are considered as powerful tools to provide next-generation immunoassays (Sang et al., 2015; Dinarelli et al., 2016). The advantages of these antibodies, e.g., ease of production in *E. coli* and its reproducibility, have made them much popular than conventional antibodies (Frenzel et al., 2017; Sun et al., 2018). More importantly, this single-chain format of antibodies can be easily fused with other proteins, such as detector enzymes, e.g., alkaline phosphatase (AP), resulting in formation of antibodies with multiple functions which can increase both the reaction rate and the sensitivity of diagnostic assays. In fact, the design of ELISA-based scFv-AP fusion proteins helps simultaneous use of two properties, namely binding specificity of antibody and AP enzyme activity. For instance, application of scFv-AP fusion proteins for different targets such as ractopamine (Dong et al., 2012), aflatoxin (Rangnoi et al., 2011), and glycocholic acid (Cui et al., 2018) have been described.

The following are examples of new diagnostic methods developed for detection of *C. difficile*. Moreover, various other diagnostic platforms based on rAb technology are well discussed.

### Enzyme-linked immunosorbent assay

One of the most favorite immunoassay techniques is ELISA. Due to its high performance, low cost, high speed and possibility to test a large number of samples simultaneously, ELISA-based kits became the standard technique for diagnosis of various agents (Mollarasouli et al., 2019). Developing toxin ELISAs as a rapid test that is based on mAbs or pAbs has always attracted much attention. For example, development of double-antibody sandwich (ds) ELISA by using mAb targeting the receptor binding region of TcdB (anti-CDB3) produced by hybridoma technologies was reported by Chen et al. which was regarded as an effective method for reliable diagnosis of CDI by detecting TcdB in diarrhea stools (Chen et al., 2015b).

Additionally, rAbs can be used as an alternative attractive strategy to mAbs and pAbs. Successful application of scFv antibodies in various ELISA platforms is reported in many studies; for instance, the development of ELISA based on phage-displayed scFv antibodies was reported for detecting *B. melitensis* (Hayhurst et al., 2003), IFN-γ antigen (Yang et al., 2018), cowpea chlorotic dwarf virus (Raeisi et al., 2020), citrus tristeza virus (Raeisi et al., 2022b; Chen et al., 2015b), the protein components of type three secretion system of *Xanthomons citri* (Raeisi et al., 2019), *B. thuringiensis* (Dong et al., 2018) and so on. Moreover, several publications have reported the development of diagnostic VHH-based ELISA assays for rapid diagnosis of specific targets. Detection of influenza H5N1 virus (Zhu et al., 2014a), *B. thuringiensis* (Wang et al., 2014), *Listeria monocytogenes* (Tu et al., 2015), and ochratoxin A (Zhang et al., 2019) are few examples of VHHs application in ELISA platforms.

The application of rAbs for the quantitative detection of toxins can also be a reliable approach, which costs less than conventional ELISAs. There are some reports in the literature for successful application of rAbs in detection of toxins. In this regard, scFv antibodies against toxin B of *C. difficile* were reported by Deng et al. (2003). The performance of anti-TcdB scFv fragments was shown by a sandwich ELISA and the results demonstrated that the isolated scFvs had high specificity to detect toxin B and did not have cross-reactivity with toxin B-negative *C. difficile* bacteria. Interestingly, isolated scFv fragment had higher sensitivity than the mAb and could detect a minimum of 10 ng of toxin B/well. In another study, Unger et al. (2015) evaluated the ability of anti-binary toxin sdAb fragments in blocking cytotoxic effect of the binary toxin, and results confirmed the proper function of sdAbs against this toxin; it was concluded that these sdAbs are promising new diagnostic tools for diseases associated with *C. difficile* (Unger et al., 2015).

### Chemiluminescent immunoassay

Chemiluminescent immunoassay (CLIA) is an immunoassay technique using luminescent molecule as an indicator of the analytic reaction that composes of combination of chemiluminescence techniques and immunochemical reactions (Cinquanta et al., 2017). This method is similar to other labeled immunoassay such as ELISA, except that CLIA substrates generate light emission in the presence of an enzyme, causing a more sensitive process compared to ELISA. Since CLIA technique has many advantages such as high sensitivity and specificity, rapidity, high stability of reagents, and compatibility with immunology assay protocols, it has been used to measure hormones, tumor markers, autoantibodies, and infectious disease markers (Chen et al., 2012).

Recently, use of magnetic-particle-based chemiluminescent enzyme immunoassay (CLEIA) for detection of *C. difficile* has been reported by Qi et al. (2020a). In this study, a CLEIA was developed using an anti-toxin B mAb-coated magnetic particles and anti-toxin B mAb conjugated to AP enzyme. The proposed CLEIA demonstrated high sensitivity for toxin B detection and showed a linear working range from 0.12 to 150 ng/mL with a limit of detection (LOD) of 0.47 ng/mL, which was similar to those of a commercial ELISA kit. This method could detect antigen in stool samples in less than 30 min, indicating high potential of the proposed method for rapid TcdB detection.

### Immunosensors

**Figure 3:**
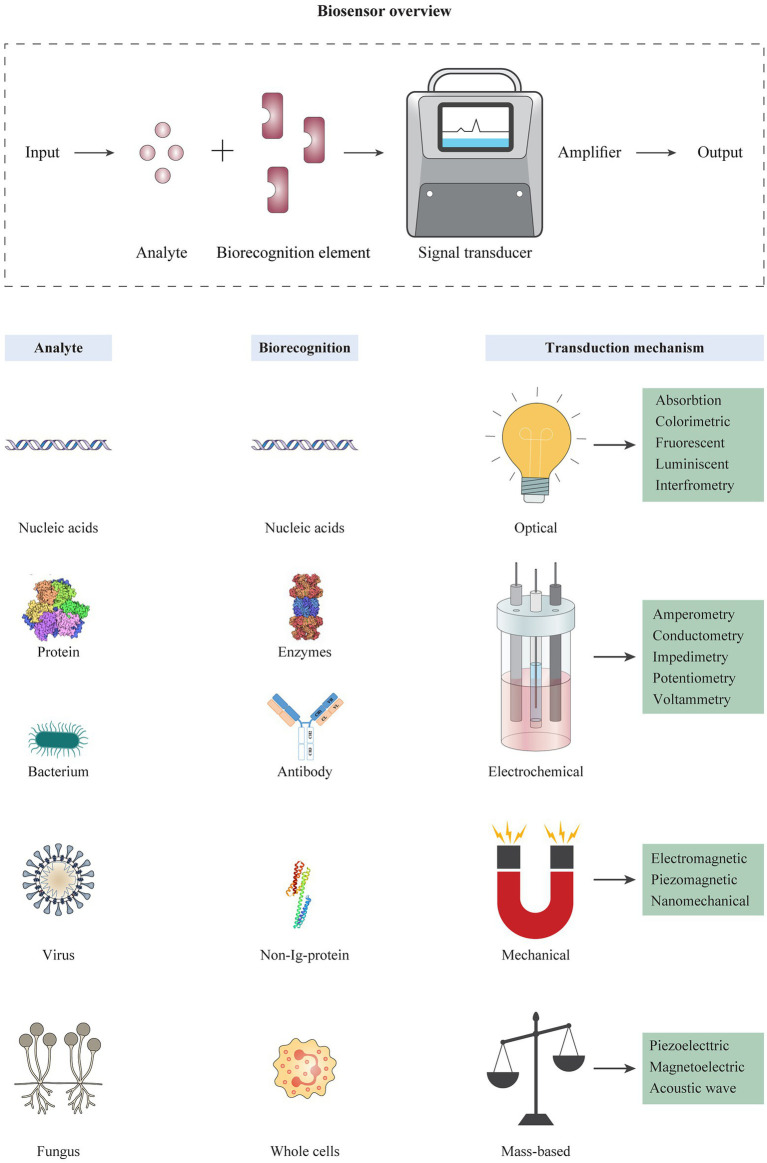
Schematic overview of biosensor platforms consisting of different types of analytes, biorecognition elements, and transduction mechanisms. The operation of a biosensor is based on detection of an analyte by a highly specific biorecognition element. Analytes usually include biomolecules such as nucleic acids, proteins, and different cells. The reaction of an analyte with the biorecognition element is transformed into an electrical, optical, or electrochemical signal by a transducer, and converted into displayable data. The different types of biorecognition elements are used in the design of biosensors, including nucleic acids, antibodies, non-long-proteins, and/or synthetic ligands. Biosensors can be categorized based on the transducing mechanism, including optical, electrochemical, mechanical, and mass-based biosensors, which each category contains several platforms.

Antibodies can be used in the design of biosensors for rapid diagnosis purposes. Biosensors are known as devices for rapid detection or determination of the concentration of biological analytes like biomolecules, biological structures or microorganisms (Mehrotra, 2016). A biosensor typically consists of a biorecognition element and a transducer (Figure 3). A biorecognition element must identify a specific analyte and interact with it. The interaction between biorecognition element and analyte changes the biosensor’s properties; these changes are converted by the transducer into a measurable signal that is a form of energy, e.g., optical, thermal, and electrical. Thus, the encounter of sensors with specific pathogens or pathogenic byproducts leads to the production of positive detection signals (Bhalla et al., 2016). Classification of biosensors is based on the type of biorecognition element (e.g., enzyme, antibody, and nucleic acid probe) or the transducing method used (e.g., piezoelectric, optical, and electrochemical; Alhadrami, 2017), and different techniques are classified like nano-or micro-technology platforms accordingly. Importantly, integration of nanotechnology with biosensor systems can augment the analytical efficiency of detection methods and provide more sensitive and accurate diagnosis (Zhu et al., 2014b; Pan et al., 2017; Cho et al., 2020). This technique helps to improve characteristics like sensitivity (e.g., low LODs), assaying time, and analytical procedures. In this regard, gold nanoparticles (AuNPs) are known as one of the most widely used nanomaterials in biomedical research and clinical imaging (Hammami et al., 2021). Biocompatibility, non-toxicity, ease of characterization, and high stability are some of the significant properties of AuNPs that promote their diverse applications (Cabuzu et al., 2015; Hu et al., 2020). So far, there are successful reports for the use of these compounds to detect various viruses and bacterial agents (Mocan et al., 2017; Singh et al., 2017).

Antibody-based sensors or immunosensors, are a popular format of biosensors that change the transducer signal when a specific antigen–antibody reaction is detected (Cho et al., 2020). Various immunosensors have been developed and they have gained much attention in biochemical analyses (Tang and Xia, 2008), clinical diagnoses (Morris, 2013), food quality control (Scognamiglio et al., 2014), and environmental monitoring (Mehrotra, 2016). Therefore, they seem to be excellent candidates for rapid and sensitive diagnosis of diseases and pathogens as far as there are many reports of their use for detection of bacteria such as *E. coli* (Park et al., 2008), *Salmonella enterica* (Cinti et al., 2017), *Salmonella typhimurium* (Fulgione et al., 2018), and *X. citri* (Haji-Hashemi et al., 2018). Immunosensors can be designed on the basis of different types of antibodies (pAbs, mAbs, and rAbs), however, most of the immunosensors are designed based on pAbs and mAbs.

Additionally, fragment antibodies such as sdAbs, scFvs, and Fabs have emerged as suitable alternatives to design biosensors (Ahmad et al., 2012). Although rAbs have several significant advantages over conventional antibodies, they are yet not fully exploited in fabrication of immunosensors. There are some reports for application of rAbs in designing various immunosensors, e.g., optical biosensors (Damborský et al., 2016; Peltomaa et al., 2018), electrochemical detection (Grewal et al., 2013), LFIA (McDonnell et al., 2010; Kim et al., 2020), and piezo-immunosensors (Shen et al., 2007). However, most sensors designed to detect *C. difficile* are based on nucleic acid probes, aptamers or mAbs, examples of which are given in Table 4, there are limited research published on biosensors that are designed based on rAbs. Below, application of different immunosensors for rapid detection of *C. difficile* are discussed.

**Table 4:** Examples of rapid detection used for *Clostridioides difficile* infection.

**Biorecognition elements**	**Target (analyte)**	**Platform**	**Detection format**	**Applicability**	**LOD**	**Response time**	**References**
Nucleic acid	Spores	Magnetic bead aggregation	LAMP coupled to PiBA	Selective media	N/A	N/A	DuVall et al. (2016)
	Toxins	Magnetic	NMR-based technology	Stool	<180 CFU/mL	45 min	Yang et al. (2017)
	TcdA/B	Magnetic	ELISA sandwich assays	Stool	1.35 𝜇g/mL	N/A	Hong et al. (2015)
	TcdA/B	MEF	Optical	Stool	10 CFU/mL	40 s	Joshi et al. (2014)
	TcdB	PEPS	Electrical impedance (PEPS) (direct detection)	Stool	150 CFU/mL	40 min	Han et al. (2019)
	TcdA/B	FMSM	Optical	Stool	1.12 ng/mL	20 min	Yang et al. (2020)
Aptamer (aptasensor)	TcdA/B, binary toxin	Magnetic Beads/SOMAmer	ELISA sandwich assays	Selective media	1 pg/mL to 300 pg/mL	N/A	Ochsner et al. (2013), Ochsner et al. (2014)
	GDH	Fuorescence emission structure-switching fluorescence signaling aptamer	Optical	Stool	1 pg/mL	60 min	Liu et al. (2018)
	TcdA	Colorimetric	Electrochemical impedance	Stool	1 pg/mL	N/A	Luo et al. (2014)
	TcdB	Colorimetric	Colorimetric assay	Stool	600 and 60 pg/mL	30 min	Liu et al. (2020)
	TcdB	Colorimetric	Colorimetric assay	Stool	5 ng/mL	30 min 60 min	Luo and Liu (2020)
	GDH	Colorimetric	Colorimetric assay	Selective media	10 𝜇g/mL	30 min	Hui et al. (2018)
Enzyme based	Specific protease, PPEP-1	Bioluminescent Sensor	Optical	Stool	10 𝜇g/mL	30 min	Ng et al. (2021)
Antibody	TcdB	Amperometric	Electrochemical	TcdB	0.3 ng/mL	30 min	Xu et al. (2014)
	TcdA/B	Impedance	Electrochemical	Stool	0.6 pg/mL	30 min	Zhu et al. (2014b)
	TcdB	DPV	Electrochemical	Stool	0.7 pg/mL	45 min	Fang et al. (2013)
	GDH	Lateral flow immunoassay	Optical, thermal	Selective media	1.0625 ng/mL 0.1328 ng/mL	N/A	Wang et al. (2016)
	TcdB	Magnetic and fluorescent particles	Automated ELISAs	Stool	45 pg/mL	30 min	Gite et al. (2018)
	Toxins	Single-molecule array technology	Automated ELISAs	Stool	45 pg/mL	N/A	Banz et al. (2018)
	TcdB	Impedance polyurethane (PU) nanospiked gold electrode	Electrochemical (DPV)	Stool	0.5 pg/mL	40 min	Cui et al. (2018)
	TcdA/B	Fluorescence	Optical	Stool	N/A	40 s	Joshi et al. (2014)
	SlpA, TcdB	SERS-based LFA	Optical	Stool	0.01 pg/mL	20 min	Hassanain et al. (2021)
	GDH, TcdB	Split-luciferase assay, luminescent	Optical	Stool	4.5 pg/mL 2 pg/mL	32 min	Adamson et al. (2022)

GDH, glutamate dehydrogenase; TcdA, toxin A; TcdB, toxin B; LAMP, microfluidic loop-mediated isothermal amplification; PiBA, product-inhibited bead aggregation; MEF, metal enhanced fluorescence; MAMEF, microwave accelerated metalenhanced fluorescence; PEPS, piezoelectric plate sensor; FMSM, fluorescent magnetic spore-based microrobot; ELISAs, enzyme-linked immunosorbent assays; PPEP-1, pro-pro endopeptidase 1; DPV, differential pulse voltammetry; SERS-based LFA, surface enhanced Raman scattering-based lateral flow assay; N/A, not available.

#### Electrochemical immunoassays

Among different types of biosensors, electrochemical immunoassays have gained much attention as a bioanalytical method. These diagnostic methods can become a promising approach with high sensitivity and specificity in the future; they have many advantages like ease of signal quantification, low cost, rapidity, high compatibility, high repeatability, high sensitivity, ease of integration, and miniaturization (Kimmel et al., 2012; Cho et al., 2020). Design of a mAb-based electrochemical immunoassay is a successful example of application of electrochemical immunosensor for detecting *C. difficile* (Fang et al., 2013). In particular, Fang and co-workers developed a simple sandwich-assay type electrochemical immunosensor to detect TcdB by using graphene oxide (GO) as a scaffold to improve the surface area to capture a large number of primary antibodies. In this system, horseradish peroxidase (HRP)-labeled secondary TcdB antibody was also introduced on the electrode surface through the unique properties of AuNPs. Coating antibodies with nanoparticles caused a large amount of antibody entering the biosensor system leading to an increase in electrochemical impedance signal, and as a result the sensitivity in diagnosis was increased, so that this system showed a linear working range from 3 pg/mL to 320 ng/mL with a LOD of 0.5 pg/mL under optimal experimental conditions, which was much lower than that of other conventional methods like ELISA. More importantly, high specificity of the produced electrochemical immunosensor for detecting TcdB in stool samples, indicated that application of this immunosensor can be a promising option in clinical diagnosis of CDI.

Application of AuNPs coated by specific antibodies to detect *C. difficile* or its toxins has been also reported by Zhu et al. (2014b). In this work, an electrochemical impedance immunosensor based on AuNPs coated with anti-toxin sdAb antibodies for detecting both TcdA and TcdB was designed (Zhu et al., 2014b). The results showed LODs of 0.61 and 0.60 pg/mL for TcdA and TcdB, respectively, that are indicative of high sensitivity of this method. Evaluation of immunosensor efficiency for detecting toxins in stool samples showed that the designed biosensor can be potentially used in clinical applications. These promising results prove that the use of AuNPs in immunosensors led to an excellent performance with high sensitivity. However, this method requires a relatively complex procedure that is not easily available.

In another study, Cui et al. (2018) developed a label-free electrochemical biosensor for TcdB detection. In this work, sdAb isolated against TcdB was used for preparing polyurethane (PU) electrodes with nanospiked gold electrode-based label-free electrochemical immunosensor. Signal amplification in this method was six times as high as flat PU gold electrode-based immunosensor. Based on these results, the PU nanospiked gold electrode-based immunosensor can be an excellent option for rapid detection of TcdB especially when low cost and simple processing is expected (Cui et al., 2018). Recently, the use of silica nanoparticles in designing electrochemical biosensor has led to sensitive and specific detection; this technique was able to sensitively detect bacteria in five minutes by cyclic voltammetry measurements, and interestingly, this device could detect other microorganisms with minor modifications within its features (Mathelie-Guinlet et al., 2019), and can be considered as a potential diagnostic platform in future.

#### Optical biosensors

In addition to electrochemical immunosensor, optical biosensors are a popular form of detection methods and known as a powerful alternative to conventional analytical techniques, particularly for their small size, high sensitivity, and cost effectiveness (Damborský et al., 2016). Diagnosis made by optical biosensors is based on sensitive detection of photon emission from dyes and molecules excited by light. Hence, fluorescent probes are the most widely used probes in these biosensors that can emit photons after interacting with specific targets such as antigens, antibodies, or genomic material. The fluorescence emission helps to increase the sensitivity, thus, the results obtained by these methods are more reliable than conventional detection methods such as microscopy or enzyme-based detection. These biosensors are widely reported for detecting bacteria, such as *S. typhimurium* (Seo et al., 1999), *E. coli* (Riangrungroj et al., 2019), and *S. enterica* (Quintela et al., 2019).

Generally, optical biosensors have been applied in different platforms. In this regard, the application of MultiPath immunoassay technology that uses non-magnified digital imaging to count molecular targets labeled microscopic fluorescent particles has been reported by Gite et al. (2018). In this work, a novel MultiPath immunoassay technology was developed for highly sensitive detection of *C. difficile* toxin B antigen based on anti-TcdB mAbs, which are conjugated to magnetic and fluorescent particles through a carboxyl linkage (EDC/NHS chemistry; Gite et al., 2018). This technology counts and images target-specific magnetic and fluorescent particles banded together by anti-TcdB in minimally processed stool samples. This technology can efficiently detect CDI due to its advantages including minimal sample preparation, rapid diagnosis, and requiring simple optical equipment. This study demonstrates that the designed immunosensor has an equivalent performance to cytotoxicity assay in terms of sensitivity for toxin B detection, so that the results showed LOD of 45 pg/mL with 97% sensitivity; 98.3% specificity; and 98.2% accuracy for the MultiPath TcdB test, which was similar to cytotoxicity assays and significantly better than on-market EIAs. In addition, this method shows superiority to NAATs because it directly detects the toxins secreted by vegetative *C. difficile* cells even at very low concentrations, while the NAATs detects the toxin in dormant spores which do not cause disease, thus, false positive results can be avoided. Recently, the use of single-molecule array (Simoa) technology was described for ultrasensitive detection of toxins (Song et al., 2015). Simoa technology, also known as digital ELISA, is based on efficient capture, labeling and detection of single protein molecules on paramagnetic beads in arrays of femtoliter-sized wells. This method has a very low LOD, and it is about 1,000-fold more sensitive than conventional ELISA (Rissin et al., 2010). There is no report on the use of specific anti-*C. difficile* rAbs using this technology and only the design of Simoa technology based on mAbs was reported for CDI diagnosis. The development of Simoa technology for detecting TcdA and TcdB by Song et al. (2015) showed that this method can detect all strains producing toxins in the stool specimens with LODs of 0.45 and 1.50 pg/mL for TcdA and TcdB, respectively (Song et al., 2015). In another work, Sandlund et al. (2018) introduced an automated single-molecule counting technology for detecting *C. difficile* toxin A and B (Sandlund et al., 2018) in stool samples with a low LOD of 2.0 pg/mL for TcdA and 0.7 pg/mL for TcdB, which was similar to Simoa technology; however, the dependence of both methods on fluorescent labeling complicates and limits their operations and use.

### Lateral flow immunoassays

Recently, researchers have made undeniable efforts to develop assays that do not rely on laboratory equipment. LFIA as one of such examples is a rapid technique which has attracted a lot of attention. Today, LFIA is widely used for various monitoring and diagnostic purposes, particularly for on-site use in veterinary to serve as analytical tests for a range of biochemical analytes, for medical and food safety purposes, home-pregnancy test (Koczuła and Gallotta, n.d.), human immunodeficiency virus (HIV) diagnosis (Nguyen et al., 2009), and SARS-CoV-2 detection (Moshe et al., 2021). The assay is performed on a sheet-shaped matrix, e.g., cellulose, containing freely labeled antibodies with a color or the fluorescent mark. The assay implements where an analyte interacts with a detection reagent and an immobilized capture reagent. Several types of antibodies (mAb, pAb, rAb, HRP-conjugated, AP-conjugated, etc.) are applied in LFIAs. Notably, LFIAs are known as a suitable approach to implement on-site and they are also compatible with already available commercial tests. Due to their customer-friendly and low-cost features, they would be potentially applied in diagnostic platforms in future (Liu et al., 2021). Application of these devices has many advantages as they are fast, easy to use, portable, and they do not require external equipment (Koczula and Gallotta, 2016), leading to the popularity of these biosensors among researchers. Currently, application of LFIA methods for determination of harmful substances in food is being investigated. The use of LFIAs to detect *Enterococcus faecalis*, *Staphylococcus aureus* (Wang et al., 2017a), *L. monocytogenes* (Wang et al., 2017b), *Shigella* (Wang et al., 2016a), and *Leptospirosis* (Nurul Najian et al., 2016) has been successful so far. Combination of LFIA methods with nanoparticles, including AuNPs (Sun et al., 2015; Fu et al., 2017; Jiang et al., 2017), fluorescent-microsphere-derived ICA (F-ICA), and time-resolved Eu/Tb (III) nanoparticles ICA (TRF-ICA) (Tang et al., 2017; Zhang et al., 2017) was suggested in biomarkers designing. Furthermore, application of nanoparticles such as gold in LFIA tests causes a thermal contrast that leads to increased sensitivity of detection systems. In a recent study, the sensitivity of visual or colorimetric readers was improved up to 8-fold for *C. difficile* (reduced from 1.0625 to 0.1328 ng/mL), thus, thermal contrast readers would receive much attention in future (Wang et al., 2016b).

In addition to lateral-flow assays, latex agglutination techniques (LAT) allow for naked-eye detection. LAT assays are based on the use of latex particles bound to antibodies, so that the antigen-binding sites are exposed and they can bind the target antigen. The binding of antibody to antigen leads to a lattice formation of the latex particles which appears as a visible agglutination. These types of techniques are rapid, easy to perform, inexpensive, fast and they do not require special equipment. The use of particles which change in color when aggregated, e.g., AuNPs, makes the visual detection possible in these techniques. In this context, rapid visual detection of bacteria such as *M. tuberculosis* (Bhaskar et al., 2004) and *E. coli* (Huang et al., 2001) was documented. Integration of this technique with nanoparticles like AuNPs increases the speed of this method in a way that target antigens can be detected in less than 30 min (Koczula and Gallotta, 2016). There is only one report on the use of antibody-latex agglutination test in detection of *C. difficile*, in which positive latex results were confirmed by cytotoxicity assay. Accordingly, this test can be an acceptable diagnostic tool for screening *C. difficile* toxins (Shahrabadi et al., 1984), which can be considered a simple and affordable diagnostic tool for clinical use.

Recently, the results of research on quantum dots (QD) have revealed that they are one of the best candidates for development of novel detection methods (Matea et al., 2017). QDs are very small nanoparticles with strong fluorescence properties that act as semi-conductors. The use of QDs in the tools developed for diagnosis of different diseases has been described (Zhao and Zeng, 2015). These diagnostic methods are semi-quantitative or quantitative and have many advantages such as being simple, fast, and easy, and they require only visual inspection. Additionally, QD technology has advantages over organic chemical fluorescent groups, such as higher quantum yield, wide absorption cross-sections, excellent optical reliability, and adjustable specification of fluorescent transmission (Dubertret et al., 2002).

Use of QDs in *C. difficile* detection has been reported; Qi et al. (2020) developed a LFIA using an anti-TcdB mAb coupled to quantum dot nanobeads (QDNBs). The LOD of QDNBs-LFA in the fecal samples was 0.297 ng/mL that was consistent with those of a commercial ELISA kit. Moreover, concerning sensitivity, QDNBs-LFA showed good correlations with clinical diagnosis. Thus, the performance of QDNBs in this diagnostic system was highly applicable for designing portable and rapid on-the-spot platforms (Qi et al., 2020b). Overall, the use of rAbs can be suggested to increase the efficiency of these biosensors (Sharma et al., 2016; Lara and Perez-Potti, 2018), and should not be ignored in future studies.

## Discussion

The current status of CDI diagnosis is still challenging. The available laboratory tests have remained confined and revealed a high frequency of inconsistency in detecting *C. difficile* colonization or diagnosis of suspected CDI patients (McDonald et al., 2018). Additionally, an accurate and early diagnosis of CDI is essential for optimal patient care, timely infection control, pharmacological treatment, and preventing the spread of infection (Gerding et al., 2016; Gateau et al., 2018; Peng et al., 2018). Therefore, the achievement of timely and accurate diagnostic assays with high sensitivity and specificity will play an increasingly pivotal role in more efficient disease management. Today, the development of diagnostic tools based on antibodies has received great attention. Some properties of antibodies, such as their high affinity and specific binding to target molecules, make them reliable therapeutic/diagnostic tools (Ahmad et al., 2012; Sharma et al., 2016). This has led to the introduction of numerous antibody-based diagnostic kits so far, however, obtaining antibodies with specificity, sensitivity, and high affinity has always been a challenge facing researchers (Hammers and Stanley, 2014). Recently, some studies have focused on the application of antibodies for CDI diagnosis; and further research is still being conducted in this area (Fang et al., 2013; Davies et al., 2015; Qi et al., 2020). Most of the research has been done on the production of antibodies using the hybridoma technology, which has been applied successfully in numerous cases, while it has many shortcomings, such as being time-consuming and inefficient in generating antibodies toward highly-conserved antigens, self-antigens, sensitive, and toxic antigens (Parray et al., 2020). In these cases, it is recommended to use alternative methods like non-antibody binding proteins (or protein scaffolds) and *in vitro* antibody display technologies (Alfaleh et al., 2020; Gebauer and Skerra, 2020). In contrast to monoclonal antibodies, protein scaffolds are single polypeptide chain structures without disulfide bridges or post-translational modifications that show high solubility and stability and can allow the selection for affinity, stability, and enzymatic activity (Simeon and Chen, 2018; Gebauer and Skerra, 2020). Additionally, *in vitro* antibody display technologies have many advantages, including the ability to be genetically modified to increase selectivity, specificity, and sensitivity, ease of production in *E. coli*, and reproducibility (Frenzel et al., 2017; Chiu et al., 2019; Raeisi et al., 2022a). Hence, various studies have suggested the use of phage display-derived antibodies for diagnostic purposes (Chamorro and Merkoçi, 2016; Mohd Ali et al., 2021). Innovative developments based on antibodies (e.g. various biosensor platforms) can provide rapid, reliable, and sensitive detection capability, which help the accurate diagnosis of specific targets. Importantly, due to the increase in public health concerns about nosocomial CDI, designing immunosensors can be proposed for rapid detection and disease management (Zhu et al., 2014b). Additionally, the combination of nanotechnology and various biosensor platforms has attracted a lot of attention as an excellent approach for developing fast, highly, sensitive and specific devices for the diagnosis of bacterial and viral infections (Prasad, 2014; Park et al., 2016), which can improve the quality and precision of disease diagnosis (Castillo et al., 2020; Younis et al., 2020).

Application of rAb fragments (scFv, sdAb, etc.) to develop immunosensors or immunoassays has several distinct advantages, the most important of them is that rAb fragments can be readily produced *in vitro*. In addition, rAbs are easily formatted through genetic engineering or chemical conjugation for coupling antibodies to the sensors used to detect antigens. These advantages in the case of antibodies that have limited *in vivo* production, especially anti-toxins, are more highlighted. Another advantage of rAb antibodies is that in scFv-based label-free immunosensors, for example, piezo-immunosensors, a high-affinity rAb is sufficient to detect the antigen, thus no antigen-specific secondary antibody (i.e., detecting) is required (Shen et al., 2005). In fact, the use of rAb in these biosensors simplifies antigen detection because in some cases, the generation of just one antigen-specific antibody is difficult, let alone two. Furthermore, chemical modification of a sensor’s surface under controlled conditions can facilitate correct orientation (e.g., on AuNPs) of rAb fragments at high density, which leads to improved avidity and sensitivity of the assay (Gandhi et al., 2018). In many sensing transducers and imaging technologies, rAb antibodies can be used instead of traditional whole antibodies, including quartz crystal microbalance (QCM), cyclic voltammetry (CV), surface plasmon resonance (SPR), and many other detection techniques (Zeng et al., 2012). These techniques can provide a tool with high sensitivity and specificity to detect antigens in complex samples like feces and blood. Moreover, a QCM based on rAbs can readily sense a change in mass on the sensor’s surface, so that antibody concentration on the immunosensor surface can be easily determined to ensure that the same concentration of antibody is used every time and this helps enhance inter-assay reproducibility. This feature is not available for most traditional immunoassays (e.g., ELISAs) (Zeng et al., 2012). Interestingly, reducing the distance between donors and acceptors in optical biosensors increases their efficiency (Damborský et al., 2016), thus the use of small-sized antibodies like rAbs can be considered as a way to increase the efficiency of these biosensors (Sharma et al., 2016; Lara and Perez-Potti, 2018). So far, the application of rAbs in various diagnostic platforms, such as ELISA, LFIA, nanoparticles, and microfluidics, has been reported (Goodchild et al., 2005; Zhang et al., 2012). It seems that due to the desirable properties of rAbs, they can be employed for designing newer diagnostic tools in the future.

## Conclusion

Presently, the precise and effective diagnosis of CDI is based on a multistep approach. Thus, the application of rapid and accurate diagnostic tools can be a key step in the management of CDI. Previous studies have demonstrated that rAbs possess high affinity and specificity for the detection of various targets and can be considered as reliable diagnostic tools in CDI management. Moreover, *in vitro* antibody production can be cost-effective compared with the conventional antibodies. This is particularly remarkable for developing novel and rapid detection tests such as biosensors. The effectiveness of rAbs can be enhanced by genetic engineering that would allow designing high performance diagnostic techniques and reducing the assay costs. Therefore, antibody generation by rAb technologies will provide an attractive platform for current and future diagnostic purposes and can be the future trend of research for designing ultrasensitive methods for CDI diagnosis.

## Author contributions

HR was involved in literature review, writing of the original draft, and figures illustration. MA was involved in literature review. AY was involved in conceptualization, preparing the draft of the manuscript, reviewing, and editing. HAA and MRZ were involved in revising the manuscript. All authors contributed to the article and approved the submitted version.

## Funding

This study was financially supported by a research grant (no. RIGLD 1138, IR.SBMU.RIGLD.REC.1399.051) from the Foodborne and Waterborne Diseases Research Center, Research Institute for Gastroenterology and Liver Diseases, Shahid Beheshti University of Medical Sciences, Tehran, Iran.

## Conflict of interest

The authors declare that the research was conducted in the absence of any commercial or financial relationships that could be construed as a potential conflict of interest.

## Publisher’s note

All claims expressed in this article are solely those of the authors and do not necessarily represent those of their affiliated organizations, or those of the publisher, the editors and the reviewers. Any product that may be evaluated in this article, or claim that may be made by its manufacturer, is not guaranteed or endorsed by the publisher.

## Abbreviations

AP: Alkaline phosphatase
AAD: Antibiotic-associated diarrhea
CCNA: Cell-culture cytotoxicity neutralization assay
CLIA: Chemiluminescent immunoassay
Cwps: Cell wall protein
*C. difficile*: *Clostridioides difficile*
CDI: *C. difficile* infection
CROPs: Combined repetitive oligopeptide structures
CV: Cyclic voltammetry
EIA: Enzyme immunoassay
ELISA: Enzyme-linked immunosorbent assay
EMA: European Medicines Agency
FliC: Flagellin;
FliD: Flagellin filament cap protein
Fabs: Fragment antigen binding domains
FDA: Food and Drug Administration
GI tract: Gastrointestinal tract
GTD: Glucosyltransferase domain
AuNPs: Gold nanoparticles
LFIAs: Lateral flow immunoassays
LOD: Limit of detection
mAbs: Monoclonal antibodies
NAATs: Nucleic acid amplification tests
PCR: Polymerase chain reaction
pAbs: Polyclonal antibodies
PU: Polyurethane
PMC: Pseudomembranous colitis
QD: Quantum dots
QDNBs: Quantum dot nanobeads
QCM: Quartz crystal microbalance
rAbs: Recombinant antibodies
SARS-CoV-2: Severe acute respiratory syndrome coronavirus 2
SLP: S-layer proteins
scFv: Single-chain fragment variable
sdAb: Single-domain antibodies
SPR: Surface plasmon resonance
TC: Toxigenic culture
VH: Variable regions of heavy domain
VL: Variable regions of light domain
ZIKV: Zika virus

## References

[ref1] Adamson H. Ajayi M. O. Gilroy K. E. McPherson M. J. Tomlinson D. C. Jeuken L. J. C. (2022). Rapid quantification of C. difficile glutamate dehydrogenase and toxin B (TcdB) with a NanoBiT Split-luciferase assay. Anal. Chem. 94, 8156–8163. doi: 10.1021/acs.analchem.1c05206, PMID: 35634999 PMC9201815

[ref2] Ahangarzadeh S. Bandehpour M. Kazemi B. (2017). Selection of single-chain variable fragments specific for mycobacterium tuberculosis ESAT-6 antigen using ribosome display. Iran. J. Basic Med. Sci. 20, 327–333. doi: 10.22038/ijbms.2017.8363, PMID: 28392906 PMC5378971

[ref3] Ahmad A. Yeap S. K. Ali A. Ho W. Y. Alitheen N. Hamid M. (2012). scFv antibody: principles and clinical application. Clin. Dev. Immunol. 2012:980250. doi: 10.1155/2012/980250 22474489 PMC3312285

[ref4] Alcalá L. Sánchez-Cambronero L. Catalán M. P. Sánchez-Somolinos M. Peláez M. T. Marín M. (2008). Comparison of three commercial methods for rapid detection of Clostridium difficile toxins a and B from fecal specimens. J. Clin. Microbiol. 46, 3833–3835. doi: 10.1128/JCM.01060-08, PMID: 18784313 PMC2576614

[ref5] Alfaleh M. A. Alsaab H. O. Mahmoud A. B. Alkayyal A. A. Jones M. L. Mahler S. M. (2020). Phage display derived monoclonal antibodies: from bench to bedside. Front. Immunol. 11, 497–508. doi: 10.3389/fimmu.2020.01986, PMID: 32983137 PMC7485114

[ref6] Alhadrami H. (2017). Biosensors: classifications, medical applications and future prospective. Biotechnol. Appl. Biochem. 65, 497–508. doi: 10.1002/bab.1621 29023994

[ref7] Alibeiki M. Golchin M. Tabatabaei M. (2020). Development of a double-recombinant antibody sandwich ELISA for quantitative detection of epsilon toxoid concentration in inactivated Clostridium perfringens vaccines. BMC Vet. Res. 16:361. doi: 10.1186/s12917-020-02572-4, PMID: 32993643 PMC7525996

[ref8] Angela Chiew Wen C. N. Choong Y. S. Lim T. (2016). Phage display-derived antibodies: application of recombinant antibodies for diagnostics. Front. Immunol. 2016:e0198. doi: 10.3389/fimmu.2020.0198

[ref9] Arimoto J. Horita N. Kato S. Fuyuki A. Higurashi T. Ohkubo H. (2016). Diagnostic test accuracy of glutamate dehydrogenase for Clostridium difficile: systematic review and meta-analysis. Sci. Rep. 6:29754. doi: 10.1038/srep29754, PMID: 27418431 PMC4945925

[ref10] Ascoli C. A. Aggeler B. (2018). Overlooked benefits of using polyclonal antibodies. BioTechniques 65, 127–136. doi: 10.2144/btn-2018-0065, PMID: 30089399

[ref11] Azimirad M. Jo Y. Kim M. S. Jeong M. Shahrokh S. Asadzadeh Aghdaei H. (2022). Alterations and prediction of functional profiles of gut microbiota after fecal microbiota transplantation for Iranian recurrent Clostridioides difficile infection with underlying inflammatory bowel disease: a pilot study. J. Inflamm. Res. 15, 105–116. doi: 10.2147/JIR.S338212, PMID: 35023946 PMC8747792

[ref12] Azimirad M. Krutova M. Yadegar A. Shahrokh S. Olfatifar M. Aghdaei H. A. (2020b). Clostridioides difficile ribotypes 001 and 126 were predominant in Tehran healthcare settings from 2004 to 2018: a 14-year-long cross-sectional study. Emerg. Microb. Infect. 9, 1432–1443. doi: 10.1080/22221751.2020.1780949, PMID: 32520657 PMC7473134

[ref13] Azimirad M. Noori M. Raeisi H. Yadegar A. Shahrokh S. Asadzadeh Aghdaei H. (2021). How does COVID-19 pandemic impact on incidence of Clostridioides difficile infection and exacerbation of its gastrointestinal symptoms? Front. Med. (Lausanne). 8:775063. doi: 10.3389/fmed.2021.775063, PMID: 34966759 PMC8710593

[ref14] Azimirad M. Yadegar A. Gholami F. Shahrokh S. Asadzadeh Aghdaei H. Ianiro G. (2020a). Treatment of recurrent Clostridioides difficile infection using fecal microbiota transplantation in Iranian patients with underlying inflammatory bowel disease. J. Inflamm. Res. 13, 563–570. doi: 10.2147/JIR.S265520, PMID: 32982371 PMC7509309

[ref15] Badger V. Ledeboer N. Graham M. B. Edmiston J. C. (2012). Clostridium difficile: epidemiology, pathogenesis, management, and prevention of a recalcitrant healthcare-associated pathogen. JPEN J. Parenter. Enteral Nutr. 36, 645–662. doi: 10.1177/0148607112446703 22577120

[ref16] Baghani A. Mesdaghinia A. Kuijper E. J. Aliramezani A. Talebi M. Douraghi M. (2020). High prevalence of Clostridiodes diffiicle PCR ribotypes 001 and 126 in Iran. Sci. Rep. 10:4658. doi: 10.1038/s41598-020-61604-z, PMID: 32170182 PMC7070088

[ref17] Bakir M. Babich J. Aftab N. Dean C. Lambrecht R. Ott R. (1993). C-erbB2 protein overexpression in breast cancer as a target for PET using iodine-124-labeled monoclonal antibodies. J. Nuclear Med. 33, 2154–2160. 1460508

[ref18] Balsalobre-Arenas L. Alarcón-Cavero T. (2017). Rapid diagnosis of gastrointestinal tract infections due to parasites, viruses, and bacteria. Enferm. Infecc. Microbiol. Clin. 35, 367–376. doi: 10.1016/j.eimc.2017.01.002 28238506 PMC7103346

[ref19] Balsells E. Filipescu T. Kyaw M. H. Wiuff C. Campbell H. Nair H. (2016). Infection prevention and control of Clostridium difficile: a global review of guidelines, strategies, and recommendations. J. Glob. Health 6:020410. doi: 10.7189/jogh.06.020410, PMID: 28028434 PMC5140074

[ref20] Banz A. Lantz A. Riou B. Foussadier A. Miller M. A. Davies K. A. (2018). Sensitivity of single-molecule Array assays for detection of Clostridium difficile toxins in comparison to conventional laboratory testing algorithms. J. Clin. Microbiol. 56. doi: 10.1128/JCM.00452-18, PMID: 29898996 PMC6062787

[ref21] Barbut F. Surgers L. Eckert C. Visseaux B. Cuingnet M. Mesquita C. (2014). Does a rapid diagnosis of Clostridium difficile infection impact on quality of patient management? Clin. Microbiol. Infect. 20, 136–144. doi: 10.1111/1469-0691.12221, PMID: 23565919

[ref22] Bazan J. Calkosinski I. Gamian A. (2012). Phage display—a powerful technique for immunotherapy. Hum. Vaccin. Immunother. 8, 1817–1828. doi: 10.4161/hv.21703 22906939 PMC3656071

[ref23] Berry S. K. Rust S. Caceres C. Irving L. Bartholdson Scott J. Tabor D. E. (2022). Phenotypic whole-cell screening identifies a protective carbohydrate epitope on *Klebsiella pneumoniae*. MAbs 14:2006123. doi: 10.1080/19420862.2021.2006123, PMID: 34923908 PMC8726669

[ref24] Bhalla N Jolly P Formisano N Estrela P. (2016). Introduction to biosensors. Essays biochem. 60, 1–8. doi: 10.1042/EBC20150001 27365030 PMC4986445

[ref25] Bhaskar S. Banavaliker J. Hanif M. (2004). Large-scale validation of a latex agglutination test for diagnosis of tuberculosis. FEMS Immunol. Med. Microbiol. 39, 235–239. 10.1016/S0928-8244(03)00232-3 14642308

[ref26] Bockstaele F. Holz J.-B. Revets H. (2000). “The development of nanobodies for therapeutic applications,” in Current Opinion in Investigational Drugs, vol. 2009 (London), 1212–1224. 19876789

[ref27] Burke K. E. Lamont J. T. (2014). Clostridium difficile infection: a worldwide disease. Gut Liver. 8, 1–6. doi: 10.5009/gnl.2014.8.1.1, PMID: 24516694 PMC3916678

[ref28] Burnham C.-A. Carroll K. (2013). Diagnosis of Clostridium difficile infection: an ongoing conundrum for clinicians and for clinical laboratories. Clin. Microbiol. Rev. 26, 604–630. doi: 10.1128/CMR.00016-13, PMID: 23824374 PMC3719497

[ref29] Burnham C-A Dubberke E Kociolek L Polage C Riley T. (2015). Clostridium difficile—Diagnostic and Clinical Challenges. Clin. Chem. 62, 310–314. doi: 10.1373/clinchem.2015.243717. 26656133

[ref30] Byrne H. Conroy P. Whisstock J. O’Kennedy R. (2013). A tale of two specificities: bispecific antibodies for therapeutic and diagnostic applications. Trends Biotechnol. 31, 621–632. doi: 10.1016/j.tibtech.2013.08.007. 24094861 PMC7114091

[ref32] Cabuzu D. Cirja A. Puiu R. Grumezescu A. M. (2015). Biomedical applications of gold nanoparticles. Curr. Top. Med. Chem. 15, 1605–1613. doi: 10.2174/1568026615666150414144750 25877087

[ref33] Castillo L. Vega Baudrit J. Lopretti M. (2020). Biosensors for the detection of bacterial and viral clinical pathogens. Sensors 20:6926. doi: 10.3390/s20236926, PMID: 33291722 PMC7730340

[ref34] Castro-Córdova P. Mora-Uribe P. Reyes-Ramírez R. Cofré-Araneda G. Orozco-Aguilar J. Brito-Silva C. (2021). Entry of spores into intestinal epithelial cells contributes to recurrence of Clostridioides difficile infection. Nat. Commun. 12:1140. doi: 10.1038/s41467-021-21355-5, PMID: 33602902 PMC7893008

[ref35] Chamorro A. Merkoçi A. (2016). Nanobiosensors in diagnostics. Nano:3. doi: 10.1177/1849543516663574 PMC5998262 29942385

[ref36] Chandrasekaran R. Lacy D. B. (2017). The role of toxins in Clostridium difficile infection. FEMS Microbiol. Rev. 41, 723–750. doi: 10.1093/femsre/fux048, PMID: 29048477 PMC5812492

[ref37] Chen X. Gentili M. Hacohen N. Regev A. (2021). A cell-free nanobody engineering platform rapidly generates SARS-CoV-2 neutralizing nanobodies. Nat. Commun. 12:5506. doi: 10.1038/s41467-021-25777-z, PMID: 34535642 PMC8448731

[ref38] Chen W. Jie W. Chen Z. Jie X. Huang-Xian J. (2012). Chemiluminescent immunoassay and its applications. Chin. J. Anal. Chem. 40, 3–10. doi: 10.1016/S1872-2040(11)60518-5

[ref39] Chen W. Liu W. E. Li Y. M. Luo S. Zhong Y. M. (2015b). Preparation and preliminary application of monoclonal antibodies to the receptor binding region of Clostridium difficile toxin B. Mol. Med. Rep. 12, 7712–7720. doi: 10.3892/mmr.2015.4369, PMID: 26459027

[ref40] Chen S. Sun C. Wang H. Wang J. (2015a). The role of rho GTPases in toxicity of Clostridium difficile toxins. Toxins (Basel). 7, 5254–5267. doi: 10.3390/toxins7124874, PMID: 26633511 PMC4690124

[ref41] Cheng J.-W. Xiao M. Kudinha T. Xu Z.-P. Sun L.-Y. Hou X. (2015). The role of glutamate dehydrogenase (GDH) testing assay in the diagnosis of Clostridium difficile infections: a high sensitive screening test and an essential step in the proposed laboratory diagnosis workflow for developing countries like China. PLoS One 10:e0144604. doi: 10.1371/journal.pone.0144604, PMID: 26659011 PMC4676637

[ref42] Chiari E. Weiss W. Simon M. Kiessig S. Pulse M. Brown S. (2021). Oral immunotherapy with human secretory IgA improves survival in the hamster model of Clostridioides difficile infection. J. Infect. Dis. 224, 1394–1397. doi: 10.1093/infdis/jiab087, PMID: 33588433 PMC8557658

[ref43] Chiu M. Goulet D. Teplyakov A. Gilliland G. (2019). Antibody structure and function: the basis for engineering therapeutics. Antibodies 8:55. doi: 10.3390/antib8040055, PMID: 31816964 PMC6963682

[ref44] Cho I.-H. Kim D. Park S. (2020). Electrochemical biosensors: perspective on functional nanomaterials for on-site analysis. Biomaterials. Research:24. 10.1186/s40824-019-0181-y PMC7001310 32042441

[ref45] Chung S.-Y. Schöttelndreier D. Tatge H. Fuehner V. Hust M. Beer L.-A. (2018). The conserved Cys-2232 in Clostridioides difficile toxin B modulates receptor binding. Front. Microbiol. 9:2314. doi: 10.3389/fmicb.2018.02314, PMID: 30416488 PMC6212469

[ref46] Cinquanta L. Fontana D. E. Bizzaro N. (2017). Chemiluminescent immunoassay technology: what does it change in autoantibody detection? Auto-immunity High. 8:9. doi: 10.1007/s13317-017-0097-2, PMID: 28647912 PMC5483212

[ref47] Cinti S. Volpe G. Piermarini S. Delibato E. Palleschi G. (2017). Electrochemical biosensors for rapid detection of foodborne salmonella: a critical overview. Sensors 17:1910. doi: 10.3390/s17081910, PMID: 28820458 PMC5579882

[ref48] Cornely O. A. Miller M. A. Louie T. J. Crook D. W. Gorbach S. L. (2012). Treatment of first recurrence of Clostridium difficile infection: fidaxomicin versus vancomycin. Clin. Infect. Dis. 55, S154–S161. doi: 10.1093/cid/cis462, PMID: 22752865 PMC3388030

[ref49] Cui X. Vasylieva N. Shen D. Barnych B. Yang J. He Q. (2018). Biotinylated single-chain variable fragment-based enzyme-linked immunosorbent assay for Glycocholic acid. Analyst 143, 2057–2065. doi: 10.1039/C7AN02024D, PMID: 29629470 PMC6449042

[ref50] Damborský P. Švitel J. Katrlík J. (2016). Optical biosensors. Essays in. Biochemistry 60, 91–100. doi: 10.1042/EBC20150010 PMC4986466 27365039

[ref51] Davies K. Berry C. Morris K. A. Smith R. Young S. Davis T. (2015). Comparison of the Vidas C. difficile GDH automated enzyme-linked fluorescence immunoassay (ELFA) with another commercial enzyme immunoassay (EIA) (Quik Chek-60), two selective media, and a PCR assay for gluD for detection of Clostridium difficile in fecal samples. J. Clin. Microbiol. 53, 1931–1934. 25788549 10.1128/JCM.00649-15 PMC4432072

[ref52] Deng X. Nesbit L. Morrow K. (2003). Recombinant single-chain variable fragment antibodies directed against Clostridium difficile toxin B produced by use of an optimized phage display system. Clin. Diagn. Lab. Immunol. 10, 587–595. PMID: 12853390 10.1128/CDLI.10.4.587-595.2003 PMC164272

[ref53] Depestel D. D. Aronoff D. M. (2013). Epidemiology of Clostridium difficile infection. J. Pharm. Pract. 26, 464–475. doi: 10.1177/0897190013499521, PMID: 24064435 PMC4128635

[ref54] Di Bella S. Ascenzi P. Siarakas S. Petrosillo N. Di Masi A. (2016). Clostridium difficile toxins A and B: insights into pathogenic properties and Extraintestinal effects. Toxins. 8:134. doi: 10.3390/toxins8050134, PMID: 27153087 PMC4885049

[ref55] Dinarelli S. Girasole M. Kasas S. Longo G. (2016). Nanotools and molecular techniques to rapidly identify and fight bacterial infections. J. Microbiol. Methods 138, 72–81. doi: 10.1016/j.mimet.2016.01.005 26806415

[ref56] Dong S. Bo Z. Zhang C. Z. Feng J. Liu X. (2018). Screening for single-chain variable fragment antibodies against multiple Cry1 toxins from an immunized mouse phage display antibody library. Appl. Microbiol. Biotechnol. 102, 3363–3374. doi: 10.1007/s00253-018-8797-8, PMID: 29484477

[ref57] Dong J.-X. Li Z. Lei H. Sun Y.-M. Ducancel F. Boulain J.-C. (2012). Development of a single-chain variable fragment-alkaline phosphatase fusion protein and a sensitive direct competitive chemiluminescent enzyme immunoassay for detection of ractopamine in pork. Anal. Chim. Acta 736, 85–91. doi: 10.1016/j.aca.2012.05.033, PMID: 22769009

[ref58] Dubberke E. R. Olsen M. A. (2012). Burden of Clostridium difficile on the healthcare system. Clin. Infect. Dis. 55, S88–S92. doi: 10.1093/cid/cis335, PMID: 22752870 PMC3388018

[ref59] Dubertret B. Skourides P. Norris D. Noireaux V. Brivanlou A. Libchaber A. (2002). *In vivo* imaging of quantum dots encapsulated in phospholipid micelles. Science (New York, N.Y.) 298, 1759–1762. doi: 10.1126/science.1077194, PMID: 12459582

[ref60] DuVall J. A. Cabaniss S. T. Angotti M. L. Moore J. H. Abhyankar M. Shukla N. (2016). Rapid detection of Clostridium difficile via magnetic bead aggregation in cost-effective polyester microdevices with cell phone image analysis. Analyst 141, 5637–5645. doi: 10.1039/C6AN00674D, PMID: 27460478

[ref61] Eastwood K. Else P. Charlett A. Wilcox M. (2009). Comparison of nine commercially available Clostridium difficile toxin detection assays, a real-time PCR assay for C. difficile tcdB, and a glutamate dehydrogenase detection assay to cytotoxin testing and cytotoxigenic culture methods. J. Clin. Microbiol. 47, 3211–3217. doi: 10.1128/JCM.01082-09, PMID: 19710274 PMC2756932

[ref62] Eze P. Balsells E. Kyaw M. H. Nair H. (2017). Risk factors for Clostridium difficile infections - an overview of the evidence base and challenges in data synthesis. J. Glob. Health 7:010417. doi: 10.7189/jogh.07.010417, PMID: 28607673 PMC5460399

[ref63] Fan G. Wang Z. Hao M. Li J. (2015). Bispecific antibodies and their applications. J. Hematol. Oncol. 8:130. doi: 10.1186/s13045-015-0227-0, PMID: 26692321 PMC4687327

[ref64] Fang Y.-S. Wang H.-Y. Wang L.-S. Wang J.-F. (2013). Electrochemical immunoassay for procalcitonin antigen detection based on signal amplification strategy of multiple nanocomposites. Biosens. Bioelectron. 51, 310–316. doi: 10.1016/j.bios.2013.07.035 23978454

[ref65] Fouladi M. Sarhadi S. Tohidkia M. Fahimi F. Samadi N. Sadeghi J. (2019). Selection of a fully human single domain antibody specific to helicobacter pylori urease. Appl. Microbiol. Biotechnol. 103, 3407–3420. doi: 10.1007/s00253-019-09674-6, PMID: 30810777

[ref66] Frenzel A. Kügler J. Helmsing S. Meier D. Schirrmann T. Hust M. (2017). Designing human antibodies by phage display. Transfus. Med. Hemother. 44, 312–318. doi: 10.1159/000479633 29070976 PMC5649246

[ref67] Fu X. Chu Y. Zhao K. Deng A. (2017). Ultrasensitive detection of the β-adrenergic agonist brombuterol by a SERS-based lateral flow immunochromatographic assay using flower-like gold-silver core-shell nanoparticles. Microchim. Acta 184, 1711–1719. doi: 10.1007/s00604-017-2178-3

[ref68] Fulgione A. Cimafonte M. Della Ventura B. Iannaccone M. Ambrosino C. Capuano F. (2018). QCM-based immunosensor for rapid detection of salmonella typhimurium in food. Sci. Rep. 8. doi: 10.1038/s41598-018-34285-y 30382128 PMC6208438

[ref69] Furuya-Kanamori L. Marquess J. Yakob L. Riley T. V. Paterson D. L. Foster N. F. (2015). Asymptomatic Clostridium difficile colonization: epidemiology and clinical implications. BMC Infect. Dis. 15:516. doi: 10.1186/s12879-015-1258-4, PMID: 26573915 PMC4647607

[ref70] Gandhi S. Banga I. Maurya P. K. Eremin S. A. (2018). A gold nanoparticle-single-chain fragment variable antibody as an immunoprobe for rapid detection of morphine by dipstick. RSC Adv. 8, 1511–1518. doi: 10.1039/C7RA12810J, PMID: 35540925 PMC9077121

[ref71] Gateau C. Couturier J. Coia J. Barbut F. (2018). How to: diagnose infection caused by Clostridium difficile. Clin. Microbiol. Infect. 24, 463–468. doi: 10.1016/j.cmi.2017.12.005 29269092

[ref72] Gebauer M. Skerra A. (2020). Engineered protein scaffolds as next-generation therapeutics. Annu. Rev. Pharmacol. Toxicol. 60, 391–415. doi: 10.1146/annurev-pharmtox-010818-021118, PMID: 31914898

[ref73] Gerding D. N. File T. M. McDonald L. C. (2016). Diagnosis and treatment of Clostridium difficile infection (CDI). Infect. Dis. Clin. Pract. 24, 3–10. doi: 10.1097/IPC.0000000000000350, PMID: 29348706 PMC5769958

[ref74] Gite S. Archambault D. Cappillino M. Cunha D. Dorich V. Shatova T. (2018). A rapid, accurate, single molecule counting method detects Clostridium difficile toxin B in stool samples. Sci. Rep. 8. doi: 10.1038/s41598-018-26353-0 29849171 PMC5976643

[ref75] Goodchild S. Love T. Hopkins N. Mayers C. (2005). Engineering antibodies for biosensor technologies. Adv. Appl. Microbiol. 58C, 185–226. doi: 10.1016/S0065-2164(05)58006-7 16543034

[ref76] Grewal Y Shiddiky M Gray S Weigel K Cangelosi G Trau M. Label-free Electrochemical Detection of an Entamoeba histolytica Antigen using Cell-free Yeast-scFv Probes (Chemical Communications, Cambridge). (2013). 10.1039/c2cc38882k PMC3564640 23329132

[ref77] Haji-Hashemi H. Norouzi P. Safarnejad M. R. Larijani B. Habibi M. M. Raeisi H. (2018). Sensitive electrochemical immunosensor for citrus bacterial canker disease detection using fast Fourier transformation square-wave voltammetry method. J. Electroanal. Chem. 820, 111–117. doi: 10.1016/j.jelechem.2018.04.062

[ref78] Hammami I. Alabdallah N. M. Jomaa A. A. Kamoun M. (2021). Gold nanoparticles: synthesis properties and applications. J. King Saud University 33:101560. doi: 10.1016/j.jksus.2021.101560

[ref79] Hammerling M. J. Fritz B. R. Yoesep D. J. Kim D. S. Carlson E. D. Jewett M. C. (2020). *In vitr*o ribosome synthesis and evolution through ribosome display. Nat. Commun. 11:1108. doi: 10.1038/s41467-020-14705-2, PMID: 32111839 PMC7048773

[ref80] Hammers C. Stanley J. (2014). Antibody phage display: technique and applications. J. Invest. Dermatol. 134:e17. doi: 10.1038/jid.2013.521 PMC3951127 24424458

[ref81] Han S. Soylu M. C. Kirimli C. E. Wu W. Sen B. Joshi S. G. (2019). Rapid, label-free genetic detection of enteropathogens in stool without genetic isolation or amplification. Biosens. Bioelectron. 130, 73–80. doi: 10.1016/j.bios.2019.01.025, PMID: 30731348 PMC6469511

[ref82] Hassanain W. Spoors J. Johnson C. Faulds K. Keegan N. (2021). Rapid ultra-sensitive diagnosis of clostridium difficile infection using a SERS-based lateral flow assay. Analyst 146, 4495–4505. doi: 10.1039/D1AN00726B, PMID: 34184680

[ref83] Hayhurst A. Happe S. Mabry R. Koch Z. Iverson B. (2003). Isolation and expression of recombinant antibody fragment to the biological warfare pathogen *Brucella melitensis*. J. Immunol. Methods 276, 185–196. doi: 10.1016/S0022-1759(03)00100-5, PMID: 12738372

[ref84] Hong K. L. Maher E. Williams R. M. Sooter L. J. (2015). In vitro selection of a single-stranded DNA molecular recognition element against Clostridium difficile toxin B and sensitive detection in human fecal matter. J Nucleic Acids. 2015:808495, 1–12. doi: 10.1155/2015/808495 25734010 PMC4334984

[ref85] Høydahl L. S. Nilssen N. R. Gunnarsen K. S. Pré M. F. Iversen R. Roos N. (2016). Multivalent pIX phage display selects for distinct and improved antibody properties. Sci. Rep. 6:39066. doi: 10.1038/srep39066, PMID: 27966617 PMC5155289

[ref86] Hu X. Zhang Y. Ding T. Liu J. Zhao H. (2020). Multifunctional Gold nanoparticles: a novel nanomaterial for various medical applications and biological activities. Front. Bioeng. Biotechnol. 13:8. doi: 10.3389/fbioe.2020.0099 PMC7438450 32903562

[ref87] Huang Y. H. Chang H.-C. Chang T. (2001). Development of a latex agglutination test for rapid identification of Escherichia coli. Eur. J. Clin. Microbiol. Infect. Dis. 20, 97–103. doi: 10.1007/PL00011250, PMID: 11305479

[ref88] Huang S. Feng L. An G. Zhang X. Zhao Z. Han R. (2018). Ribosome display and selection of single-chain variable fragments effectively inhibit growth and progression of microspheres in vitro and in vivo. Cancer Sci. 109, 1503–1512. doi: 10.1111/cas.13574, PMID: 29575477 PMC5980252

[ref89] Huang H. Weintraub A. Fang H. Nord C. E. (2009). Comparison of a commercial multiplex real-time PCR to the cell cytotoxicity neutralization assay for diagnosis of clostridium difficile infections. J. Clin. Microbiol. 47, 3729–3731. doi: 10.1128/JCM.01280-09, PMID: 19741082 PMC2772590

[ref90] Hui C. Y. Liu M. Li Y. Brennan J. D. (2018). A paper sensor printed with multifunctional bio/nano materials. Angew. Chem. 130, 4639–4643. doi: 10.1002/ange.201712903 29504183

[ref91] Humphries R. M. Uslan D. Z. Rubin Z. (2013). Performance of Clostridium difficile toxin enzyme immunoassay and nucleic acid amplification tests stratified by patient disease severity. J. Clin. Microbiol. 51, 869–873. doi: 10.1128/JCM.02970-12, PMID: 23269736 PMC3592059

[ref92] Hussack G. Arbabi Ghahroudi M. Faassen H. Songer J. Ng K. Mackenzie R. (2011). Neutralization of Clostridium difficile toxin a with single-domain antibodies targeting the cell receptor binding domain. J. Biol. Chem. 286, 8961–8976. doi: 10.1074/jbc.M110.198754, PMID: 21216961 PMC3058971

[ref93] Hussack G. Ryan S. Faassen H. Rossotti M. Mackenzie C. (2018). Neutralization of Clostridium difficile toxin B with VHH-fc fusions targeting the delivery and CROPs domains. PLoS One 13:e0208978. doi: 10.1371/journal.pone.0208978, PMID: 30540857 PMC6291252

[ref94] Hwang Y.-C. Lu R.-M. Su S.-C. Chiang P.-Y. Ko S.-H. Ke F.-Y. (2022). Monoclonal antibodies for COVID-19 therapy and SARS-CoV-2 detection. J. Biomed. Sci. 29:1. doi: 10.1186/s12929-021-00784-w, PMID: 34983527 PMC8724751

[ref95] Jarrige V. Nieuwenhuis J. Son J. Martens M. Vissers J. (2011). A fast intraoperative PTH point-of-care assay on the Philips handheld magnotech system. Deutsche Gesellschaft für Chirurgie. 396, 337–343. doi: 10.1007/s00423-010-0733-z, PMID: 21170757 PMC3044233

[ref96] Jensen L. Kilstrup M. Karatt-Vellatt A. McCafferty J. Laustsen A. (2018). Basics of antibody phage display technology. Toxins. 10:236. doi: 10.3390/toxins10060236 29890762 PMC6024766

[ref97] Jiang X. Li X. Yang Z. Eremin S. Zhang X. Y. (2017). Evaluation and optimization of three different immunoassays for rapid detection Zearalenone in fodders. Food Anal. Methods 10, 256–262. doi: 10.1007/s12161-016-0576-5

[ref98] Joshi L. T. Mali B. L. Geddes C. D. Baillie L. (2014). Extraction and sensitive detection of toxins a and B from the human pathogen Clostridium difficile in 40 seconds using microwave-accelerated metal-enhanced fluorescence. PLoS One 9:e104334. doi: 10.1371/journal.pone.0104334, PMID: 25162622 PMC4146460

[ref99] Kandalaft H. Hussack G. Aubry A. van Faassen H. Guan Y. Arbabi-Ghahroudi M. (2015). Targeting surface-layer proteins with single-domain antibodies: a potential therapeutic approach against Clostridium difficile-associated disease. Appl. Microbiol. Biotechnol. 99, 8549–8562. doi: 10.1007/s00253-015-6594-1, PMID: 25936376 PMC4768215

[ref100] Kelly C. R. Fischer M. Allegretti J. R. LaPlante K. Stewart D. B. Limketkai B. N. (2021). ACG clinical guidelines: prevention, diagnosis, and treatment of Clostridioides difficile infections. Am. J. Gastroenterol. 116, 1124–1147. doi: 10.14309/ajg.0000000000001278, PMID: 34003176

[ref101] Kim H. Kim W. H. Kim M. Jeong S. H. Lee K. (2014). Evaluation of a rapid membrane enzyme immunoassay for the simultaneous detection of glutamate dehydrogenase and toxin for the diagnosis of Clostridium difficile infection. ALM 34, 235–239. doi: 10.3343/alm.2014.34.3.235 PMC3999323 24790912

[ref102] Kim H.-Y. Lee J.-H. Kim M. Park S. Choi M. Lee W. (2020). Development of a SARS-CoV-2-specific biosensor for antigen detection using scFv-fc fusion proteins. Biosens. Bioelectron. 175:112868. doi: 10.1016/j.bios.2020.112868 33281048 PMC7703470

[ref103] Kimmel D. W. LeBlanc G. Meschievitz M. E. Cliffel D. E. (2012). Electrochemical sensors and biosensors. Anal. Chem. 84, 685–707. doi: 10.1021/ac202878q, PMID: 22044045 PMC3264821

[ref104] Koczula K. M. Gallotta A. (2016). Lateral flow assays. Essays Biochem. 60, 111–120. doi: 10.1042/EBC20150012, PMID: 27365041 PMC4986465

[ref105] Koczuła K Gallotta A. Lateral Flow Assays. 10.1042/EBC20150012 PMC4986465 27365041

[ref106] Köhler G. Milstein C. (1975). Continuous cultures of fused cells secreting antibody of predefined specificity. Nature 256, 495–497. doi: 10.1038/256495a0, PMID: 1172191

[ref107] Kuhn P. Fuehner V. Unkauf T. Moreira G. Frenzel A. Miethe S. (2016). Recombinant antibodies for diagnostics and therapy against pathogens and toxins generated by phage display. Proteomics Clin. Appl. 10, 922–948. doi: 10.1002/prca.201600002, PMID: 27198131 PMC7168043

[ref108] Kunamneni A. Ogaugwu C. Bradfute S. Durvasula R. (2020). Ribosome display technology: applications in disease diagnosis and control. Antibodies (Basel) 9. doi: 10.3390/antib9030028 PMC7551589 32605027

[ref109] Kunamneni A. Ye C. Bradfute S. B. Durvasula R. (2018). Ribosome display for the rapid generation of high-affinity Zika-neutralizing single-chain antibodies. PLoS One 13:e0205743. doi: 10.1371/journal.pone.0205743, PMID: 30444865 PMC6239285

[ref110] Lagoutte P. Lugari A. Elie C. Potisopon S. Donnat S. Mignon C. (2019). Combination of ribosome display and next generation sequencing as a powerful method for identification of affibody binders against β-lactamase CTX-M15. New Biotechnol. 50, 60–69. doi: 10.1016/j.nbt.2019.01.004, PMID: 30634000

[ref111] Lara S. Perez-Potti A. (2018). Applications of nanomaterials for Immunosensing. Biosensors 8:104. doi: 10.3390/bios8040104, PMID: 30388865 PMC6316038

[ref112] Lessa F. C. Mu Y. Bamberg W. M. Beldavs Z. G. Dumyati G. K. Dunn J. R. (2015). Burden of Clostridium difficile infection in the United States. N. Engl. J. Med. 372, 825–834. doi: 10.1056/NEJMoa1408913, PMID: 25714160 PMC10966662

[ref113] Li R. Kang G. Hu M. Huang H. (2019). Ribosome display: a potent display technology used for selecting and evolving specific binders with desired properties. Mol. Biotechnol. 61, 60–71. doi: 10.1007/s12033-018-0133-0, PMID: 30406440

[ref114] Liu M. Wang J. Chang Y. Zhang Q. Chang D. Hui C. Y. (2020). In vitro selection of a DNA aptamer targeting degraded protein fragments for biosensing. Angew. Chem. Int. Ed. Engl. 59, 7706–7710. doi: 10.1002/anie.202000025, PMID: 32155319

[ref115] Liu M. Yin Q. Brennan J. D. Li Y. (2018). Selection and characterization of DNA aptamers for detection of glutamate dehydrogenase from Clostridium difficile. Biochimie 145, 151–157. doi: 10.1016/j.biochi.2017.08.015, PMID: 28882627

[ref116] Liu Y. Zhan L. Qin Z. Sackrison J. Bischof J. C. (2021). Ultrasensitive and highly specific lateral flow assays for point-of-care diagnosis. ACS Nano 15, 3593–3611. doi: 10.1021/acsnano.0c10035, PMID: 33607867

[ref117] Lu R.-M. Hwang Y.-C. Liu I. J. Lee C.-C. Tsai H.-Z. Li H.-J. (2020). Development of therapeutic antibodies for the treatment of diseases. J. Biomed. Sci. 27:1. doi: 10.1186/s12929-019-0592-z, PMID: 31894001 PMC6939334

[ref118] Luo P. Liu Y. (2020). Detection of toxin B of Clostridium difficile based on immunomagnetic separation and aptamer-mediated colorimetric assay. Lett. Appl. Microbiol. 71, 596–604. doi: 10.1111/lam.13383, PMID: 32920822

[ref119] Luo P. Liu Y. Xia Y. Xu H. Xie G. (2014). Aptamer biosensor for sensitive detection of toxin a of Clostridium difficile using gold nanoparticles synthesized by Bacillus stearothermophilus. Biosens. Bioelectron. 54, 217–221. doi: 10.1016/j.bios.2013.11.013, PMID: 24287407

[ref120] Matea C.-T. Mocan T. Tabaran F. Pop T. A. Mosteanu O. Puia I. (2017). Quantum dots in imaging, drug delivery and sensor applications. Int. J. Nanomedicine 12, 314–320. doi: 10.1016/j.snb.2019.03.144, PMID: 28814860 PMC5546783

[ref121] Mathelie-Guinlet M. Cohen-Bouhacina T. Gammoudi I. Martin A. Beven L. Delville M. H. (2019). Silica nanoparticles-assisted electrochemical biosensor for the rapid, sensitive and specific detection of *Escherichia coli*. Sensors Actuat. B Chemical 12, 5421–5431. doi: 10.2147/IJN.S138624

[ref122] McDonald L. C. Gerding D. N. Johnson S. Bakken J. S. Carroll K. C. Coffin S. E. (2018). Clinical practice guidelines for Clostridium difficile infection in adults and children: 2017 update by the Infectious Diseases Society of America (IDSA) and Society for Healthcare Epidemiology of America (SHEA). Clin. Infect. Dis. 66, e1–e48. doi: 10.1093/cid/cix1085, PMID: 29462280 PMC6018983

[ref123] McDonnell B. Hearty S. Finlay W. O’Kennedy R. (2010). A high-affinity recombinant antibody permits rapid and sensitive direct detection of myeloperoxidase. Anal. Biochem. 410, 1–6. doi: 10.1016/j.ab.2010.09.039 20920456

[ref124] Mehrotra P. (2016). Biosensors and their applications – a review. Journal of Oral biology and craniofacial. Research 6, 153–159. doi: 10.1016/j.jobcr.2015.12.002 PMC4862100 27195214

[ref125] Merrigan M. Venugopal A. Roxas J. Anwar F. Mallozzi M. Roxas B. (2013). Surface-layer protein a (SlpA) is a major contributor to host-cell adherence of Clostridium difficile. PLoS One 8:e78404. doi: 10.1371/journal.pone.0078404, PMID: 24265687 PMC3827033

[ref126] Mocan T. Matea C. Pop T. A. Mosteanu O. Buzoianu A. Puia I. (2017). Development of nanoparticle-based optical sensors for pathogenic bacterial detection. J. Nanobiotechnol. 15.doi: 10.1186/s12951-017-0260-y PMC5374694 28359284

[ref127] Mohd Ali M. R. Sum J. S. Nn A. B. Choong Y. S. Amdan A. Amran F. (2021). Development of monoclonal antibodies against recombinant LipL21 protein of pathogenic Leptospira through phage display technology. Int. J. Biol. Macromol. 168, 289–300. doi: 10.1016/j.ijbiomac.2020.12.062, PMID: 33310091

[ref128] Mollarasouli F. Kurbanoglu S. Ozkan S. A. (2019). The role of electrochemical Immunosensors in clinical analysis. Biosensors 9:86. doi: 10.3390/bios9030086, PMID: 31324020 PMC6784381

[ref129] Moon H. W. Kim H. N. Hur M. Shim H. S. Kim H. Yun Y. M. (2016). Comparison of diagnostic algorithms for detecting toxigenic Clostridium difficile in routine practice at a tertiary referral Hospital in Korea. PLoS One 11:e0161139. doi: 10.1371/journal.pone.0161139, PMID: 27532104 PMC4988646

[ref130] Morris M. (2013). Fluorescent biosensors - probing protein kinase function in cancer and drug discovery. Biochim. Biophys. Acta 1834, 1387–1395. doi: 10.1016/j.bbapap.2013.01.025 23376184

[ref131] Moshe M. Daunt A. Flower B. Simmons B. Brown J. C. Frise R. (2021). SARS-CoV-2 lateral flow assays for possible use in national covid-19 seroprevalence surveys (react 2): diagnostic accuracy study. BMJ 372:n423. doi: 10.1136/bmj.n423 33653694 PMC7921617

[ref132] Ng K. K. Reinert Z. E. Corver J. Resurreccion D. Hensbergen P. J. Prescher J. A. (2021). A bioluminescent sensor for rapid detection of PPEP-1, a Clostridioides difficile biomarker. Sensors 21:7485. doi: 10.3390/s21227485, PMID: 34833562 PMC8624784

[ref133] Nguyen S. Wells S. Pau C.-P. Owen M. Dong X. LaBorde R. (2009). Rapid detection of HIV-1 p24 antigen using magnetic immuno-chromatography (MICT). J. Virol. Methods 160, 14–21. doi: 10.1016/j.jviromet.2009.04.003 19482361

[ref134] Nurul Najian A. B. Engku Nur Syafirah E. A. Ismail N. Mohamed M. Yean C. Y. (2016). Development of multiplex loop mediated isothermal amplification (m-LAMP) label-based Gold nanoparticles lateral flow dipstick biosensor for detection of pathogenic Leptospira. Anal. Chim. Acta 903, 142–148. doi: 10.1016/j.aca.2015.11.015 26709307

[ref135] Ochsner U. A. Green L. S. Gold L. Janjic N. (2014). Systematic selection of modified aptamer pairs for diagnostic sandwich assays. BioTechniques 56:125-8, 30–32-3. doi: 10.2144/000114134 24641476

[ref136] Ochsner U. A. Katilius E. Janjic N. (2013). Detection of Clostridium difficile toxins a, B and binary toxin with slow off-rate modified aptamers. Diagn. Microbiol. Infect. Dis. 76, 278–285. doi: 10.1016/j.diagmicrobio.2013.03.029, PMID: 23680240

[ref137] Oldfield E. C. I. V. Oldfield E. C. Johnson D. A. (2014). Clinical update for the diagnosis and treatment of Clostridium difficile infection. World J. Gastrointest. Pharmacol. Ther. 5, 1–26. doi: 10.4292/wjgpt.v5.i1.1, PMID: 24729930 PMC3951810

[ref138] Orrell K. E. Melnyk R. A. (2021). Large Clostridial toxins: mechanisms and roles in disease. Microbiol. Mol. Biol. Rev. 85:e0006421. doi: 10.1128/MMBR.00064-21, PMID: 34076506 PMC8483668

[ref139] Paitan Y. Miller-Roll T. Adler A. (2017). Comparative performance study of six commercial molecular assays for rapid detection of toxigenic Clostridium difficile. Clin. Microbiol. Infect. 23, 567–572. doi: 10.1016/j.cmi.2017.02.016, PMID: 28223147

[ref140] Pan M. Gu Y. Yun Y. Li M. Jin X. Wang S. (2017). Nanomaterials for electrochemical Immunosensing. Sensors (Basel). 17:1041. doi: 10.3390/s17051041, PMID: 28475158 PMC5469646

[ref141] Paredes-Sabja D. Shen A. Sorg J. A. (2014). Clostridium difficile spore biology: sporulation, germination, and spore structural proteins. Trends Microbiol. 22, 406–416. doi: 10.1016/j.tim.2014.04.003, PMID: 24814671 PMC4098856

[ref142] Park M. (2020). Surface display Technology for Biosensor Applications: a review. Sensors (Basel). 20. doi: 10.3390/s20102775 PMC7294428 32414189

[ref143] Park S. Kim H. Paek S.-H. Cho D.-W. Kim Y.-K. (2008). Enzyme-linked immuno-strip biosensor to detect Escherichia coli O157: H7. Ultramicroscopy 108, 1348–1351. doi: 10.1016/j.ultramic.2008.04.063, PMID: 18562109

[ref144] Park C. S. Lee C. Kwon O. S. (2016). Conducting Polymer Based Nanobiosensors. Polymers 8:249. doi: 10.3390/polym8070249, PMID: 30974524 PMC6432403

[ref145] Parray H. Shukla S. Samal S. Shrivastava T. Ahmed S. Sharma C. (2020). Hybridoma technology a versatile method for isolation of monoclonal antibodies, its applicability across species, limitations, advancement and future perspectives. Int. Immunopharmacol.:85. doi: 10.1016/j.intimp.2020.106639 PMC7255167 32473573

[ref146] Peltomaa R. Glahn-Martínez B. Benito-Peña E. Moreno-Bondi M. C. (2018). Optical biosensors for label-free detection of small molecules. Sensors (Basel) 18:4126. doi: 10.3390/s18124126, PMID: 30477248 PMC6308632

[ref147] Peng Z. Ling L. Stratton C. W. Li C. Polage C. R. Wu B. (2018). Advances in the diagnosis and treatment of Clostridium difficile infections. Emerg. Microb. Infect. 7:15. doi: 10.1038/s41426-017-0019-4, PMID: 29434201 PMC5837143

[ref148] Planche T. D. Davies K. A. Coen P. G. Finney J. M. Monahan I. M. Morris K. A. (2013). Differences in outcome according to Clostridium difficile testing method: a prospective multicentre diagnostic validation study of C difficile infection. Lancet Infect. Dis. 13, 936–945. doi: 10.1016/S1473-3099(13)70200-7, PMID: 24007915 PMC3822406

[ref149] Planche T. Wilcox M. (2011). Reference assays for Clostridium difficile infection: one or two gold standards? J. Clin. Pathol. 64, 1–5. doi: 10.1136/jcp.2010.080135 21118850

[ref150] Pollock N. (2015). Ultrasensitive detection and quantification of toxins for optimized diagnosis of Clostridium difficile infection. J. Clin. Microbiol. 54, 259–264. 10.1128/JCM.02419-15 PMC4733189 26659205

[ref151] Pollock N. R. Banz A. Chen X. Williams D. Xu H. Cuddemi C. A. (2019). Comparison of Clostridioides difficile stool toxin concentrations in adults with symptomatic infection and asymptomatic carriage using an ultrasensitive quantitative immunoassay. Clin. Infect. Dis. 68, 78–86. doi: 10.1093/cid/ciy415, PMID: 29788296 PMC6293008

[ref152] Prasad S. (2014). Nanobiosensors: the future for diagnosis of disease? Nanobiosens. Dis. Diagn. 3, 1–10. doi: 10.2147/NDD.S39421

[ref153] Priyanka B. Patil R. K. Dwarakanath S. (2016). A review on detection methods used for foodborne pathogens. Indian J. Med. Res. 144, 327–338. doi: 10.4103/0971-5916.198677, PMID: 28139531 PMC5320838

[ref154] Qi H. Sun Q. Ma Y. Wu P. Wang J. (2020b). Advantages of lateral flow assays based on fluorescent submicrospheres and quantum dots for Clostridium difficile toxin B detection. Toxins. 12. doi: 10.3390/toxins12110722, PMID: 33227925 PMC7699250

[ref155] Qi H. Wang Y. Wu P. Ma Y. Wang J. (2020a). Rapid and fully-automated detection of Clostridium difficile toxin B via magnetic-particle-based chemiluminescent immunoassay. Am. J. Transl. Res. 12, 4228–4236. PMID: 32913500 PMC7476116

[ref156] Quintela I. A. de Los Reyes B. G. Lin C.-S. Wu V. C. H. (2019). Simultaneous colorimetric detection of a variety of salmonella spp. in food and environmental samples by optical biosensing using oligonucleotide-Gold nanoparticles. Front. Microbiol. 10:1138. doi: 10.3389/fmicb.2019.01138, PMID: 31214132 PMC6554661

[ref157] Raeisi H. (2018). Production of polyclonal phages harbouring antibody fragment genes against *Xanthomonas citri* subsp citri using phage display technology. Appl. Entomol. Phytopathol. 85, 265–276.

[ref158] Raeisi H. Azimirad M. Nabavi-Rad A. Asadzadeh Aghdaei H. Yadegar A. Zali M. R. (2022a). Application of recombinant antibodies for treatment of Clostridioides difficile infection: current status and future perspective. Front. Immunol. 13:972930. doi: 10.3389/fimmu.2022.972930, PMID: 36081500 PMC9445313

[ref159] Raeisi H. Safarnejad M. R. Alavi S. M. Elahinia S. A. Farrokhi N. (2020). Applying the pthA effector protein of Xanthomonas citri subsp. citri for production of specific antibodies and its application for detection of infected plants. J. Plant Pathol. 102, 79–87. doi: 10.1007/s42161-019-00385-5

[ref160] Raeisi H. Safarnejad M. R. Alavi S. M. Farrokhi N. Elahinia S. A. Safarpour H. (2019). Development and molecular analyses of Xanthomonas pthA specific scFv recombinant monoclonal antibodies. Mdrsjrns. 8, 417–429.

[ref1700] Raeisi H. Safarnejad M. R. Sadeghkhani F. (2022b). A new single-chain variable fragment (scFv) antibody provides sensitive and specific detection of citrus tristeza virus. J. Virol Methods 300:114412. doi: 10.1016/j.jviromet.2021.114412 34896452

[ref161] Raeisi H. Safarnejad M. R. Moeini P. Safarpour H. Sokhansanj Y. (2020). Isolation of single-chain variable fragment (scFv) antibodies for detection of chickpea chlorotic dwarf virus (CpCDV) by phage display. Arch. Virol. 165, 2789–2798. doi: 10.1007/s00705-020-04813-1, PMID: 32970278

[ref162] Ramos C. P. Lopes E. O. Diniz A. N. Lobato F. C. F. Vilela E. G. Silva R. O. S. (2020). Evaluation of glutamate dehydrogenase (GDH) and toxin a/B rapid tests for Clostridioides (prev. clostridium) difficile diagnosis in a university hospital in Minas Gerais, Brazil. Braz. J. Microbiol. 51, 1139–1143. doi: 10.1007/s42770-020-00288-z PMC7455615 32367261

[ref163] Rangnoi K. Jaruseranee N. O’Kennedy R. Pansri P. Yamabhai M. (2011). One-step detection of aflatoxin-B1 using scFv-alkaline phosphatase-fusion selected from human phage display antibody library. Mol. Biotechnol. 49, 240–249. doi: 10.1007/s12033-011-9398-2, PMID: 21465334

[ref164] Reller M. E. Alcabasa R. C. Lema C. A. Carroll K. C. (2010). Comparison of two rapid assays for Clostridium difficile common antigen and a C difficile toxin a/B assay with the cell culture neutralization assay. Am. J. Clin. Pathol. 133, 107–109. doi: 10.1309/AJCPO3QWOU8CYGEU, PMID: 20023265

[ref165] Riangrungroj P. Bever C. S. Hammock B. D. Polizzi K. M. (2019). A label-free optical whole-cell Escherichia coli biosensor for the detection of pyrethroid insecticide exposure. Sci. Rep. 9:12466. doi: 10.1038/s41598-019-48907-6, PMID: 31462650 PMC6713742

[ref166] Rissin D. Kan C. Campbell T. Howes S. Fournier D. Song L. (2010). Single-molecule enzyme-linked immunosorbent assay detects serum proteins at subfemtomolar concentrations. Nat. Biotechnol. 28, 595–599. doi: 10.1038/nbt.1641, PMID: 20495550 PMC2919230

[ref167] Roovers R. Vosjan M. Laeremans T. Khoulati R. de Bruin R. Ferguson K. (2011). A biparatopic anti-EGFR nanobody efficiently inhibits solid tumour growth. Int. J. Cancer 129, 2013–2024. doi: 10.1002/ijc.26145, PMID: 21520037 PMC4197845

[ref168] Sandlund J. Bartolome A. Almazan A. Tam S. Biscocho S. Abusali S. (2018). Ultrasensitive detection of C. difficile toxins a and B using automated single molecule counting technology. J. Clin. Microbiol.:56. doi: 10.1128/JCM.00908-18 PMC6204678 30158195

[ref169] Sang S. Wang Y. Feng Q. Wei Y. Ji J. Zhang W. (2015). Progress of new label-free techniques for biosensors: a review. Crit. Rev. Biotechnol. 36, 1–17. doi: 10.3109/07388551.2014.991270 25608959

[ref170] Schirrmann T. Meyer T. Schütte M. Frenzel A. Hust M. (2011). Phage display for the generation of antibodies for proteome research, diagnostics and therapy. Molecules (Basel, Switzerland) 16, 412–426. doi: 10.3390/molecules16010412, PMID: 21221060 PMC6259421

[ref171] Scognamiglio V. Arduini F. Palleschi G. Rea G. (2014). Biosensing technology for sustainable food safety. TrAC Trends Anal. Chem. 62, 1–10. doi: 10.1016/j.trac.2014.07.007

[ref172] Seo K.-H. Brackett R. Hartman N. Campbell D. (1999). Development of a rapid response biosensor for detection of salmonella typhimurium. J. Food Prot. 62, 431–437. doi: 10.4315/0362-028X-62.5.431, PMID: 10340660

[ref173] Shahrabadi M. S. Bryan L. E. Gaffney D. Coderre S. E. Gordon R. Pai C. H. (1984). Latex agglutination test for detection of Clostridium difficile toxin in stool samples. J. Clin. Microbiol. 20, 339–341. doi: 10.1128/jcm.20.3.339-341.1984, PMID: 6490824 PMC271325

[ref174] Shali A. Hasannia S. Gashtasbi F. Shahangian S. Jalili S. (2018). Generation and screening of efficient neutralizing single domain antibodies (VHHs) against the critical functional domain of anthrax protective antigen (PA). Int. J. Biol. Macromol. 114, 1267–1278. doi: 10.1016/j.ijbiomac.2018.03.034, PMID: 29524493

[ref175] Sharma S. Byrne H. O'Kennedy R. (2016). Antibodies and antibody-derived analytical biosensors. Essays Biochem. 60, 9–18. doi: 10.1042/EBC20150002, PMID: 27365031 PMC4986469

[ref176] Shen Z. Stryker G. Mernaugh R. Yu L. Yan H. Zeng X. (2005). Single-Chain Fragment Variable Antibody Piezoimmunosensors. Anal. Chem. 77, 797–805. doi: 10.1021/ac048655w, PMID: 15679346 PMC2505340

[ref177] Shen Z. Yan H. Parl F. F. Mernaugh R. L. Zeng X. (2007). Recombinant antibody piezoimmunosensors for the detection of cytochrome P450 1B1. Anal. Chem. 79, 1283–1289. doi: 10.1021/ac061211a, PMID: 17297925 PMC2504758

[ref178] Shirvan A. N. Aitken R. (2016). Isolation of recombinant antibodies directed against surface proteins of Clostridium difficile. Braz. J. Microbiol. 47, 394–402. doi: 10.1016/j.bjm.2016.01.017, PMID: 26991284 PMC4874623

[ref179] Simeon R. Chen Z. (2018). In vitro-engineered non-antibody protein therapeutics. Protein Cell 9, 3–14. doi: 10.1007/s13238-017-0386-6, PMID: 28271446 PMC5777970

[ref180] Singh R. Hong S. Jang J. (2017). Label-free detection of influenza viruses using a reduced graphene oxide-based electrochemical Immunosensor integrated with a microfluidic platform. Sci. Rep. 7:42771. doi: 10.1038/srep42771, PMID: 28198459 PMC5309888

[ref181] Song L. Zhao M. Duffy D. C. Hansen J. Shields K. Wungjiranirun M. (2015). Development and validation of digital enzyme-linked immunosorbent assays for ultrasensitive detection and quantification of Clostridium difficile toxins in stool. J. Clin. Microbiol. 53, 3204–3212. doi: 10.1128/JCM.01334-15, PMID: 26202120 PMC4572538

[ref182] Stills H. F. (2012). “Chapter 11 - polyclonal antibody production.” in The Laboratory Rabbit, Guinea Pig, Hamster, and Other Rodents. eds. Suckow M. A. Stevens K. A. Wilson R. P. (Boston: Academic Press).

[ref183] Sun Z. Wang X. Qi C. Yun Y.-H. Tang Z. Liu X. (2018). Nanobody-alkaline phosphatase fusion protein-based enzyme-linked immunosorbent assay for one-step detection of Ochratoxin a in Rice. Sensors 18:4044. doi: 10.3390/s18114044, PMID: 30463338 PMC6263964

[ref184] Sun Y. Xing G. Yang J. Wang F. Deng R. Zhang G. (2015). Development of an Immunochromatographic test strip for simultaneous, qualitative and quantitative detection of Ochratoxin a and Zearalenone in cereal. J. Sci. Food Agric. 96, 3673–3678. doi: 10.1002/jsfa.7550 26612142

[ref185] Surawicz C. Brandt L. Binion D. Ananthakrishnan A. Curry S. Gilligan P. (2013). Guidelines for diagnosis, treatment, and prevention of Clostridium difficile infections. Am. J. Gastroenterol. 108, 478–498. doi: 10.1038/ajg.2013.4 23439232

[ref186] Tang X. Li P. Zhang Q. Zhang Z. Zhang W. Jiang J. (2017). Time-resolved fluorescence Immunochromatographic assay developed using two Idiotypic Nanobodies for rapid, quantitative, and simultaneous detection of aflatoxin and Zearalenone in maize and its products. Anal. Chem. 89, 11520–11528. doi: 10.1021/acs.analchem.7b02794, PMID: 28901744

[ref187] Tang D. Xia B. (2008). Electrochemical immunosensor and biochemical analysis for carcinoembryonic antigen in clinical diagnosis. Microchim. Acta 163, 41–48. doi: 10.1007/s00604-007-0918-5

[ref188] Tu Z. Qi C. Li Y. Xiong Y. Xu Y. Hu N. (2015). Identification and characterization of species-specific nanobodies for the detection of listeria monocytogenes in milk. Anal. Biochem. 493, 1–7. doi: 10.1016/j.ab.2015.09.023 26456330

[ref189] Tullila A. Nevanen T. (2017). Utilization of multi-immunization and multiple selection strategies for isolation of Hapten-specific antibodies from recombinant antibody phage display libraries. Int. J. Mol. Sci. 18:1169. doi: 10.3390/ijms18061169, PMID: 28561803 PMC5485993

[ref190] Unger M. Eichhoff A. Schumacher L. Strysio M. Menzel S. Schwan C. (2015). Selection of Nanobodies that block the enzymatic and cytotoxic activities of the binary clostridium difficile toxin CDT. Sci. Rep. 5:5. doi: 10.1038/srep07850 PMC4297958 25597743

[ref191] Valldorf B. Hinz S. C. Russo G. Pekar L. Mohr L. Klemm J. (2022). Antibody display technologies: selecting the cream of the crop. Biol. Chem. 403, 455–477. doi: 10.1515/hsz-2020-0377, PMID: 33759431

[ref192] van Prehn J. Reigadas E. Vogelzang E. H. Bouza E. Hristea A. Guery B. (2021). European Society of Clinical Microbiology and Infectious Diseases: 2021 update on the treatment guidance document for Clostridioides difficile infection in adults. Clin. Microbiol. Infect. 27, S1–S21. doi: 10.1016/j.cmi.2021.09.038, PMID: 34678515

[ref193] Viswanathan V. K. Mallozzi M. J. Vedantam G. (2010). Clostridium difficile infection: An overview of the disease and its pathogenesis, epidemiology and interventions. Gut Microbes 1, 234–242. doi: 10.4161/gmic.1.4.12706, PMID: 21327030 PMC3023605

[ref194] Wang X. Coljee V. Maynard J. (2013). Back to the future: recombinant polyclonal antibody therapeutics. Curr. Opin. Chem. Eng. 2, 405–415. doi: 10.1016/j.coche.2013.08.005, PMID: 24443710 PMC3892273

[ref195] Wang P. Guanghui L. Yan J. Hu Y. Zhang C. Z. Liu X. (2014). Bactrian camel nanobody-based immunoassay for specific and sensitive detection of Cry1Fa toxin. Toxicon 92, 186–192. doi: 10.1016/j.toxicon.2014.10.024, PMID: 25448390

[ref196] Wang Y. Li H. Wang Y. Li H. Luo L. Xu J. (2017b). Development of multiple cross displacement amplification label-based gold nanoparticles lateral flow biosensor for detection of listeria monocytogenes. Int. J. Nanomedicine 12, 473–486. doi: 10.2147/IJN.S123625, PMID: 28138243 PMC5238772

[ref197] Wang Y. Li H. Wang Y. Zhang L. Xu J. Ye C. (2017a). Loop-mediated isothermal amplification label-based Gold nanoparticles lateral flow biosensor for detection of enterococcus faecalis and Staphylococcus aureus. Front. Microbiol. 8.doi: 10.3389/fmicb.2017.00192 PMC5300967 28239371

[ref198] Wang Y. Qin Z. Boulware D. Pritt B. Sloan L. González I. (2016b). Thermal contrast amplification reader yielding 8-fold analytical improvement for disease detection with lateral flow assays. Anal. Chem. 88, 11774–11782. doi: 10.1021/acs.analchem.6b03406, PMID: 27750420

[ref199] Wang Y. Wang Y. Xu J. Ye C. (2016a). Development of multiple cross displacement amplification label-based Gold nanoparticles lateral flow biosensor for detection of Shigella spp. Front. Microbiol. 7 doi: 10.3389/fmicb.2016.01834 PMC5114309 27917160

[ref200] Willats W. (2003). Phage display: practicalities and prospects. Plant Mol. Biol. 50, 837–854. doi: 10.1023/a:1021215516430 12516857

[ref201] Wilson D. Rissin D. Kan C. Fournier D. Piech T. Campbell T. (2015). The Simoa HD-1 analyzer: a novel fully automated digital immunoassay analyzer with single-molecule sensitivity and multiplexing. J. Lab. Automat. 21, 533–547. doi: 10.1177/2211068215589580 26077162

[ref202] Xu X. Fang Y. Wang L. (2014). A label-free electrochemical Immunosensor for clostridium difficile toxin B based on one-step immobilization of Thionine in a silica matrix. Anal. Lett. 47, 2255–2265. doi: 10.1080/00032719.2014.900623

[ref203] Xu C. Zhang C. Z. Zhong J. Hu H. Luo S. Liu X. (2017). Construction of an immunized rabbit phage display library for selecting high-activity of against bacillus thuringiensis Cry1F toxin single-chain antibodies. J. Agric. Food Chem. 65, 6016–6022. doi: 10.1021/acs.jafc.7b01985, PMID: 28621534

[ref204] Yang P. Hash S. Park K. Wong C. Doraisamy L. Petterson J. (2017). Application of nuclear magnetic resonance to detect toxigenic Clostridium difficile from stool specimens. J. Mol. Diagn. 19, 230–235. doi: 10.1016/j.jmoldx.2016.09.012 28081922

[ref205] Yang Z. Schmidt D. Liu W. Li S. Shi L. Sheng J. (2014). A novel multivalent, single-domain antibody targeting TcdA and TcdB prevents fulminant Clostridium difficile infection in mice. J. Infect. Dis. 210, 964–972. doi: 10.1093/infdis/jiu196 24683195 PMC4192054

[ref206] Yang L. Zhang Y. Wang Q. Zhang L. (2020). An automated microrobotic platform for rapid detection of C. diff toxins. I.E.E.E. Trans. Biomed. Eng. 67, 1517–1527. doi: 10.1109/TBME.2019.2939419 31494540

[ref207] Yang H. Zhong Y. Wang J. Zhang Q. Li X. Ling S. (2018). Screening of a ScFv antibody with high affinity for application in human IFN-γ immunoassay. Front. Microbiol. 9:261. doi: 10.3389/fmicb.2018.00261 29563896 PMC5850876

[ref208] Yoldas O. Altindis M. Cufali D. Asik G. Kesli R. (2016). A diagnostic algorithm for the detection of Clostridium difficile-associated diarrhea. Balkan Med. J. 33, 80–86. doi: 10.5152/balkanmedj.2015.15159, PMID: 26966622 PMC4767316

[ref209] Younis S. Taj A. Zia R. Hayat H. Shaheen A. Awan F. (2020). Nanosensors for the detection of viruses. Nanosens. Smart Cities, 327–338. doi: 10.1016/B978-0-12-819870-4.00018-9

[ref210] Yücesoy M. McCoubrey J. Brown R. Poxton I. (2002). Detection of toxin production in Clostridium difficile strains by three different methods. Clin. Microbiol. Infect. 8, 413–418. doi: 10.1046/j.1469-0691.2002.00440.x, PMID: 12199851

[ref211] Zahavi D. Weiner L. (2020). Monoclonal antibodies in cancer therapy. Antibodies (Basel). 9. doi: 10.3390/antib9030034 PMC7551545 32698317

[ref212] Zeng X. Shen Z. Mernaugh R. (2012). Recombinant antibodies and their use in biosensors. Anal. Bioanal. Chem. 402, 3027–3038. doi: 10.1007/s00216-011-5569-z, PMID: 22159424 PMC5766000

[ref213] Zhang W.-J. Sui Y.-X. Budha A. Zheng J.-B. Sun X.-J. Hou Y.-C. (2012). Affinity peptide developed by phage display selection for targeting gastric cancer. World J. Gastroenterol. 18, 2053–2060. doi: 10.3748/wjg.v18.i17.2053, PMID: 22563192 PMC3342603

[ref214] Zhang X. Yu X. Wen K. Li C. Mujtaba G. Jiang H. (2017). Multiplex lateral flow immunoassays based on amorphous carbon nanoparticles for detecting three fusarium mycotoxins in maize. J. Agric. Food Chem. 65, 8063–8071. doi: 10.1016/j.jmoldx.2016.09.012 28825819

[ref215] Zhang C. Zhang Q. Tang X. Zhang W. Li P. (2019). Development of an anti-Idiotypic VHH antibody and toxin-free enzyme immunoassay for Ochratoxin a in cereals. Toxins. 11:280. doi: 10.3390/toxins11050280, PMID: 31137467 PMC6563187

[ref216] Zhao M.-X. Zeng E.-Z. (2015). Application of functional quantum dot nanoparticles as fluorescence probes in cell labeling and tumor diagnostic imaging. Nanoscale Res. Lett. 10:171. doi: 10.1186/s11671-015-0873-8, PMID: 25897311 PMC4397224

[ref217] Zhu M. Gong X. Hu Y. Ou W. Wan Y. (2014a). Streptavidin-biotin-based directional double Nanobody sandwich ELISA for clinical rapid and sensitive detection of influenza H5N1. J. Transl. Med. 12:352. doi: 10.1186/s12967-014-0352-5, PMID: 25526777 PMC4274719

[ref218] Zhu Z. Shi L. Feng H. Zhou H. S. (2014b). Single domain antibody coated gold nanoparticles as enhancer for Clostridium difficile toxin detection by electrochemical impedance immunosensors. Bioelectrochemistry 101, 153–158. doi: 10.1016/j.bioelechem.2014.10.003 PMC4706752 25460611

